# Properties of Polymer Composites Used in High-Voltage Applications

**DOI:** 10.3390/polym8050173

**Published:** 2016-04-28

**Authors:** Ilona Pleşa, Petru V. Noţingher, Sandra Schlögl, Christof Sumereder, Michael Muhr

**Affiliations:** 1 Polymer Competence Center Leoben GmbH (PCCL), Roseggerstrasse 12, Leoben 8700, Austria; sandra.schloegl@pccl.at; 2 Faculty of Electrical Engineering, Electrotechnical Material Laboratory, University Politehnica of Bucharest, Splaiul Independentei 313, Bucharest 060042, Romania; petrunot@elmat.pub.ro; 3 Institute of Energy, Transport and Environmental Management, University of Applied Science–FH Joanneum, Werk-VI-Straße 46, Kapfenberg 8605, Austria; christof.sumereder@fh-joanneum.at; 4 Institute of High Voltage Engineering and System Performance, Graz University of Technology, Inffeldgasse 18/I, Graz 8010, Austria; muhr@tugraz.at

**Keywords:** micro/nanocomposites, electrical properties, mechanical properties, thermal properties, high-voltage applications, cross-linked polyethylene, epoxy resins, numerical and analytical models, polymer/filler interface

## Abstract

The present review article represents a comprehensive study on polymer micro/nanocomposites that are used in high-voltage applications. Particular focus is on the structure-property relationship of composite materials used in power engineering, by exploiting fundamental theory as well as numerical/analytical models and the influence of material design on electrical, mechanical and thermal properties. In addition to describing the scientific development of micro/nanocomposites electrical features desired in power engineering, the study is mainly focused on the electrical properties of insulating materials, particularly cross-linked polyethylene (XLPE) and epoxy resins, unfilled and filled with different types of filler. Polymer micro/nanocomposites based on XLPE and epoxy resins are usually used as insulating systems for high-voltage applications, such as: cables, generators, motors, cast resin dry-type transformers, *etc.* Furthermore, this paper includes ample discussions regarding the advantages and disadvantages resulting in the electrical, mechanical and thermal properties by the addition of micro- and nanofillers into the base polymer. The study goals are to determine the impact of filler size, type and distribution of the particles into the polymer matrix on the electrical, mechanical and thermal properties of the polymer micro/nanocomposites compared to the neat polymer and traditionally materials used as insulation systems in high-voltage engineering. Properties such as electrical conductivity, relative permittivity, dielectric losses, partial discharges, erosion resistance, space charge behavior, electric breakdown, tracking and electrical tree resistance, thermal conductivity, tensile strength and modulus, elongation at break of micro- and nanocomposites based on epoxy resin and XLPE are analyzed. Finally, it was concluded that the use of polymer micro/nanocomposites in electrical engineering is very promising and further research work must be accomplished in order to diversify the polymer composites matrices and to improve their properties.

## 1. Introduction

In the last two decades, the design of composite materials comprising either micro-scaled or nano-scaled inorganic particles has gained increased attention in power and high-voltage engineering [1,2,3,4,5,6,7,8]. Particularly, the use of micro- and nanotechnologies offers new approaches towards improved insulation systems that operate at higher temperatures and electrical stress. Along with material performance, basic research and development of “advanced” materials in the field of polymer base composites also pursue energy-efficient and low cost manufacturing routes in order to bring new material concepts into marketable products [1].

Composite materials typically consist of two or more components that comprise significantly different physical and/or chemical properties. Due to the controlled combination of the components, new materials are obtained with distinct properties from the individual components [2]. If at least one of the components has nanometric dimensions, these materials are termed nanocomposites [3]. In Reference [3] a nanocomposite is defined as “a multiphase solid material where one of the phases has one, two or three dimensions of less than 100 nanometers (nm), or structures with repeating distances between the different phases in nanoscale that form the material”. Nanocomposites differ from traditional composites in three major aspects: (i) they contain a small amount of filler (usually less than 10 wt % *vs.* more than 50 wt % for composites); (ii) the filler size is in the range of nanometers in size (10^−9^ m *vs.* 10^−6^ m for composites) and (iii) they have tremendously large specific surface area compared to micro-sized composites [4]. Thus, nanocomposites are characterized by distinctive advantages including homogenous structure, no fiber rupture, and optical transparency, improved or unchanged processabillity [4]. Depending on the matrix material, nanocomposites can be classified in three major categories: ceramic matrix nanocomposites, metal matrix nanocomposites and polymer matrix nanocomposites [5]. In Reference [4], polymer matrix nanocomposites are considered as “polymers in which a small amount of nanometer size fillers (≤ 10 wt %) is homogeneously dispersed”.

Composite materials are typically desired to be employed instead of traditional materials due to their enhanced materials performance involving high strength, toughness, heat resistance, light weight, impermeability against gasses, thermal endurance and stability in the presence of aggressive chemicals, water and hydrocarbons, high resistance to fatigue and corrosion degradation, re-processing recyclability and less leakage of small molecules such as stabilizers, *etc.* [4]. In particular, in the field of plastic engineering, composite materials are selected as a function of Young’s modulus *versus* density or yield strength *versus* density [4]. For numerous applications in automotive, aircraft or maritime industry, light-weight materials with increased mechanical strength are preferred to be used. The present review addresses polymer based micro- and nanocomposites that are employed in high-voltage applications and gives an overview of electrical, mechanical and thermal properties of composite materials in dependence on the material structures and compositions.

In the power industry, inorganic filler (particularly aluminum nitride (AlN), boron nitride (BN), silicon dioxide or silica (SiO_2_), aluminum oxide or alumina (Al_2_O_3_), titanium oxide or titania (TiO_2_), silicon carbide (SiC) and zinc oxide (ZnO), *etc.*) are usually incorporated into electrical insulating polymers to achieve specific electrical, mechanical, and thermal properties [1,6]. As an example, the resistance of nanocomposites to partial discharges and electric treeing enables the design of new insulation systems with enhanced electrical breakdown strength. Beside electrical properties, mechanical strength as well as thermal conductivity play an important role in selected applications such as insulation systems of large electrical machines. In addition, permittivity and dissipation factor are desired to be as low as possible for electrical insulation whilst for capacitors, loss factor should be as high as possible. Flame retardancy is a property desired for cables insulation used in the radiation field in tunnels, while tracking resistance is very important for outdoor insulators [4].

**Figure 1:**
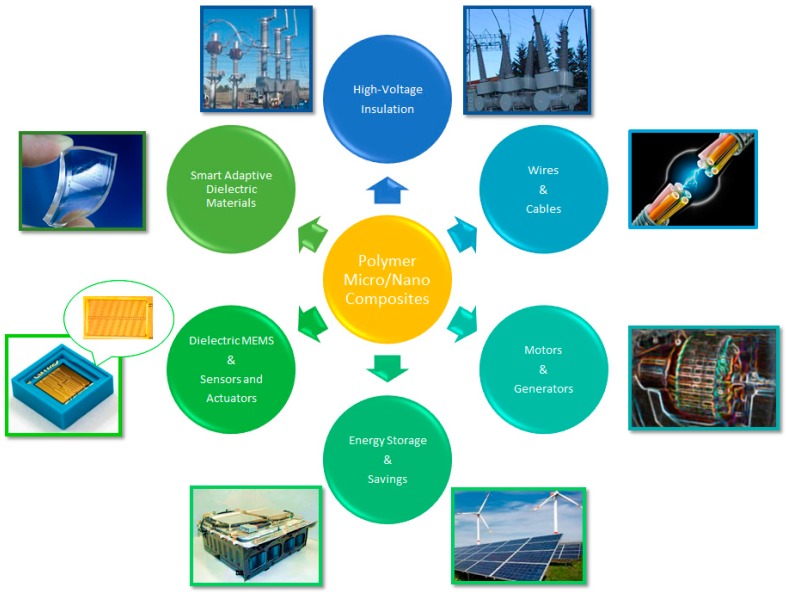
The next generation of high-voltage applications employing polymer based nanocomposites.

The present study highlights the most recent studies and results concerning micro- and nanocomposites materials used in high-voltage applications and possible future work on these materials as the distinctive advantages of polymer based (nano) composites (*i.e.*, high temperature performance, improved dielectrics, structural properties and designability) offer promising concepts for the next generation of large motors, generators, transformers and other electrical devices, such as coil forms, slot liners and multifunctional components [7] (see Figure 1).

## 2. From Micro to Nanocomposites in Electrical Engineering

**Figure 2:**
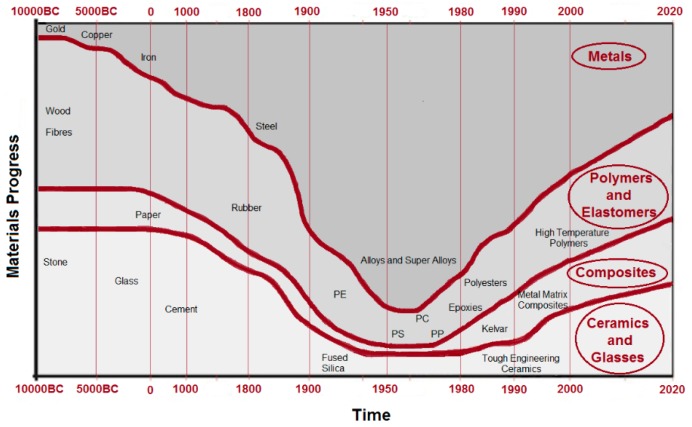
Evolution of the engineering materials (Redraw and adapted figure from [8]).

In 1987, Ashley described a perspective on advanced materials and the evolution of engineering materials (see Figure 2) [8]. It is obvious that the time scale is non-linear and in 2020, the estimation on materials usage is in a continuous increasing and the rate of change is far faster today than any previous time in history. The rapid rate of change offers opportunities that cannot be ignored by materials scientists, engineers and chemists. As a prominent example, engines efficiency increases at high operating temperatures and this requires high temperature resistant structural materials. However, new materials for rotating machines electrical insulation systems are not only faced by higher operation temperatures, but also by increased electrical, environmental and mechanical stresses. Further examples are nuclear power plants that require advanced materials for electrical equipment which are resistant to low- and high-energy radiations. In addition, developments in optical communication strongly rely on optical fibers that absorb light negligibly and on structural materials that are strong as metals and resist corrosion as plastics.

In electrical engineering, the first insulation systems were composite materials based on natural fibers of cellulose, silk, flax, cotton, wool, asbestos, sand, mica, quartz, *etc.* and natural resins derived from trees, plants, insects and petroleum deposits including pitch, shellac, rosin or linseed oil [9]. The fillers were applied as individual strands for wires and in combined forms as in nonwoven papers and woven cloths. The fact that in the early years of electrical industry, the focus was on renewable materials and trial experimentation to find systems which met minimum design criteria has to be take into account. Thus, operating temperatures, mechanical and electrical stresses were kept low to accommodate the limitations of these materials [9].

During the First World War, mica splittings were combined with bitumen or asphalts, supported on both sides by a fine grade of cellulose paper. The so-called Kraft paper was formed by muscovite mica splittings bonded with natural shellac [9]. Mica is an inorganic crystalline natural substance occurring commonly in bedrock. Chemically, mica is a complex silicate of aluminum with traces of other elements. The most employed varieties of mica are muscovite (K_2_O·3Al_2_O_3_·6SiO_2_·2H_2_O) and phlogopite (K_2_O·7MgO·Al_2_O_3_·6SiO_2_·3H_2_O). The structure of mica is complex and consists of silicon atoms layers (placed in the centers of some tetrahedrons formed by oxygen atoms) and aluminum, bounded together by oxygen atoms. Potassium atoms and hydroxyl groups (−OH) provide the connections between layers. This structure enables the flakes to be split into thin strips [10]. The thermal endurance of mica is very high. Mica starts to lose its water at a temperature of 500 °C, although some types endure even above 1100 °C. These values are more adequate for electrical machines, since the highest permitted temperatures for their parts are usually above 200 °C at the maximum [10]. Both dielectric strength and surface resistance of mica are high whilst the dielectric losses are low. Mica is characterized by a higher resistance to creepage currents and partial discharges in comparison to the best organic insulators. Taking into consideration all the properties described above, mica is almost a compulsory material in high-voltage electrical machines [10].

**Figure 3:**
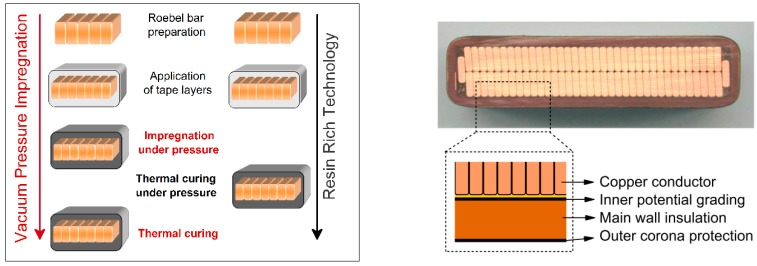
Vacuum pressure impregnation and resin rich processes in manufacturing high-voltage insulation composites of rotating machines stator bars (Redrawn and adapted figure from [13,14]).

Initially, mica was used as insulating material, in the way of small flakes and later for the manufacture of composite materials based on mica with natural (shellac, bitumen, *etc.*) and synthetic (bakelite, epoxy, polyester, *etc.*) resins used for the insulation systems of medium and high power electrical machines. Nowadays, mica is used mainly for mica paper and is composed of extremely small flakes of mica and produced in the same way as paper [11]. The composites were prepared by using a vacuum, impregnation and pressurized process, call also VPI (vacuum pressure impregnation) process and they were employed in groundwall insulation of turbine generator stator coils (see Figure 3) [9].

Micafolium insulation systems were being manufactured in the same time as the asphaltic mica systems and at the beginning, they were applied for sheet wrapping of high-voltage coils and shaped insulating parts. Park [12] synthesized epoxy resin/mica composites and estimated their electrical breakdown, in order to use the composite materials for manufacturing the insulation systems of high-voltage machines. In particular, mica particles with dimensions of 5–7 µm and different concentrations (20, 30 and 40 wt %) were applied. To reduce the composites viscosity, a plasticizer or a low molecular aliphatic epoxy was used [12]. The electrical breakdown strength (measured with a sphere-to-sphere electrodes system) was increasing by the addition of mica filler and an optimum was achieved for a mica content of 20 wt % [12]. The electrical breakdown strength of the system with an aliphatic epoxy was higher than of the system with a plasticizer [12].

The beginning of synthetic products for insulations started in 1908 with phenol-formaldehyde resins, which were used in different electrical applications. Between the 1920s and 1940s, other synthetic products were introduced in the electrical engineering industry, including alkyd resins, aniline-formaldehyde, polyvinyl chloride (PVC), urea-formaldehyde, acrylic, polystyrene (PS) and nylon and melamine-formaldehyde, glass fibers, *etc.*, which lead to an explosion of new applications in electrical insulation. During the 1940s and the 1950s, the availability of numerous types of synthetic polymers and resins increased tremendously. Polyesters and polyethylenes (PEs) were introduced in 1942, fluorocarbons and silicones in 1943, epoxies in 1947 and polyurethane (PUR), polypropylene (PP) and polycarbonate (PC) in the 1950s [9]. In the 1950s, the insulation engineers started to investigate the proliferation of new materials made from synthetic plastic films and, later, polymer fiber based nonwovens were employed for induction motors. Another major development was the replacement of solvent-borne natural and synthetic resins with solventless synthetic resins, such as polyesters, epoxy resins, silicones, acrylates, imides, blends of phenolics and other resins, which make their application more environmentally friendly and less likely to form voids within the insulation systems [9]. Historically epoxy-based composites have been widely used in both, the power as well as the microelectronics industry due to their generally superior electrical, mechanical and thermal properties along with their economical and convenient processability. In power industry, epoxy resin is still the most popular applied material for stator groundwall insulation systems. Epoxy resins with micro-scaled inorganic fillers (quartz flour) are particularly applied for dry distribution transformers and for voltage and current transformers. Previous work has demonstrated that the properties of epoxy/inorganic filler composites are governed by the chemical nature, physical structure, shape and dispersion of the inorganic filler within the epoxy matrix [1].

The composites industry began to mature in the 1970s, when improved plastic resins and reinforcing fibers (*i.e.*, Kevlar [9]) were developed and since then it has been in a continuous evolution. The first sign of “new materials” was given in the 1990s by Toyota research group that developed the first polymer nanocomposites based on clay and nylon-6 with improved thermal and mechanical performances, for timing belt covers [15].

Although the concept of “nanometric dielectrics” [16] or simply “nanodielectrics” [17] was introduced for the first time in 1994 by Lewis [16], it did not became clear how electrical insulation would benefit by the potential property changes due to nano-sized filler inclusion. Numerous studies regarding electrical phenomena in nanodielectrics, their electrical and thermal properties and the fabrication of different devices and systems with novel properties obtained due to their nanometric structures were achieved [16,18]. In 1988, Johnston and Markovitz [19] showed that some advantages could be obtained for mica-based systems used for the groundwall insulation of the form-wound generators. In 1999 Henk *et al.* [20] made similar investigations on SiO_2_ nanoparticles which are improving the voltage endurance of polymer insulation when they are dispersed in polymers. Even so, the potential application of nanodielectrics in the area of high-voltage and power engineering did not draw too much attention from researchers and material engineers until the pioneer experimental work of Nelson and Fothergill *et al.* [21,22]. A series of experimental work was performed in order to obtain a fundamental understanding of the way in which nanoparticles interact in a polymer matrix (especially, epoxy resin) to modify the dielectric properties.

Nanodielectrics analyzed by Fréchette *et al.*, in their work [17], were used to explore nanometric dielectrics and dielectrics associated with nanotechnology and to produce molecularly tailored materials [17]. Starting from these experimental results, intensive work and research has been invested on preparation, evaluation, and characterization of this new generation of materials, called nanodielectrics [23]. The interest in researching nanodielectrics materials has been increased in the last 10 years and different working groups were formed all over the over the world, as CIGRE WG D1.24 who investigated the potential of polymer nanocomposites as electrical insulation [24,25,26]. The first review articles on the nanodielectrics results was published in 2004 [4,18,27]. The studies suggested that the unique properties of polymer nanocomposites used as dielectrics in high-voltage applications are due to the interfaces, which play a key role in determining dielectric performance. In addition, several publications indicated that self-assembly is a crucial process in the formulation of nanocomposites [28,29].

**Figure 4:**
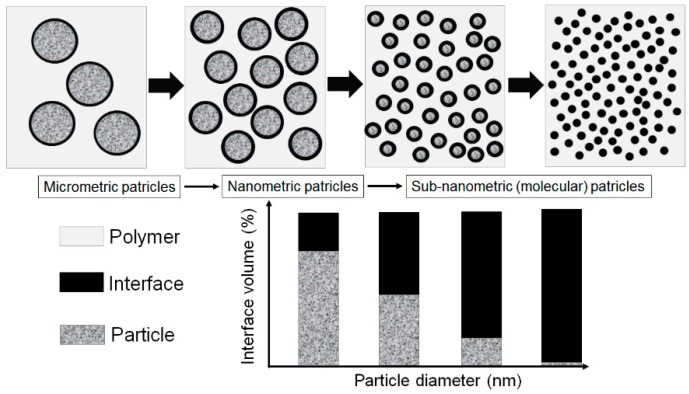
Schematic representation of the ratio particles/interfaces changes with the size of the filler (Redrawn and adapted figure from [30]).

The “interfaces” between inorganic fillers and the organic polymers (see Figure 4), such as epoxy resin systems, represent the key to understand the mechanisms and phenomena which control the properties of nanocomposites used as advanced dielectrics [1]. Therefore, interfacial control is critical for achieving good coupling between the inorganic filler and the base polymer. The present goal of the research studies in this field is to optimize these benefits and to provide a better understanding of the physical and chemical structure of the interface region [1]. In this case, there is a promise of new and enhanced properties, which are derived from the interactions between fillers and polymer matrices. Due to the complexity of nano-, meso- and micro-materials interactions, there is a large number of variables to tailor novel properties, which could be interesting for scientists and material engineers [1].

In order to describe the scientific development of micro and nanocomposites electrical features desired in power engineering, the review article is focused on the electrical, mechanical and thermal properties of the insulating materials, especially cross-linked polyethylene (XLPE) and epoxy resins, unfilled and filled with different types of particles. Polymer micro/nanocomposites based on XLPE and epoxy resins are usually used as insulating systems for high-voltage applications, such as cables, electrical machines (especially power generators and motors), dry transformers, *etc.*

## 3. Nanocomposite Used in High-Voltage Applications

Among properties enhancement, perhaps the most important property of the composites is the change in electric strength that is found when the filler particles attain nanometric dimensions [1]. Recent investigations have shown that the epoxy based nanocomposites [31] demonstrate some advantages in both mechanical and dielectric properties compared to pure resin systems and epoxy resin composites with micro-fillers at low concentration (1–10 wt %) [4,32,33]. It was found that over a wide range of frequency, the permittivity values of epoxy nanocomposites were reduced significantly compared to the base resin and epoxy micrometer-size filler at lower concentration. It was revealed that the reduction of the permittivity values strongly depends on filler type as well as filler size [33,34,35]. On the other hand, the presence of nanofillers in epoxy resin affects the space charge accumulation in polymer matrix [26,36,37,38]. The accumulation of space charge has a huge influence on dielectric properties of insulation systems. Earlier researcher in this field showed that the accumulation of space charge could affect the internal electric field which can present important local intensifications and may lead to partial discharges, electrical treeing and to an early breakdown of the insulation [31,39,40]. Consequently, it is very important to reduce space charge accumulation and its influence on dielectric behavior of insulating materials. Several works revealed that epoxy nanocomposites could accumulate less charge than neat epoxy resins [26,31]. It was also observed that epoxy nanocomposites provide faster charge dynamics, especially for negative charges [41]. Thus, it is important to study the influence of matrices and the chemical structure of fillers on the space charge accumulation in order to avoid and/or reduce their influence on the lifetime of polymer composites used in electrical engineering.

Gröpper *et al.* [42] showed that with the utilization of specially treated spherical SiO_2_ nanoparticles as part of the well-approved epoxy-mica stator winding insulation for large electrical machinery it is possible to improve significantly the properties of the high-voltage insulation system. Resistance to partial discharge erosion and electrical treeing is greatly increased and results in a longer lifetime (until electrical breakdown). In addition, the mechanical and thermal properties, which are important for stator windings of large turbine and hydro generators, showed increased values due to the application of nanocomposites [42]. The mica-based impregnating resin includes an epoxy resin/anhydride mixture and nanoparticles filler, as SiO_2_ and/or Al_2_O_3_ modified by a silanizing agent. Further, a method of producing the mica-based impregnating resin is provided, too [43]. To improve the partial discharge resistance, Gröpper *et al.* [44] have used an insulating tape comprises a mica paper and a carrier material that are glued to each other by means of an adhesive The adhesive comprises at least one nanoparticulate filler material and the insulating tape wound about the conductor is impregnated with plastic resin [44].

Nowadays, the topic of interest and technical importance in electrical power generators industry is thermal conductivity of the VPI insulation [1]. In order to improve the performance of a generator or motor, the thermal conductivity of the insulation must be improved for increased thermal power dissipation capability of the stator slot [1]. In order to obtain all these performances, the increase in thermal conductivity of insulation systems components is very important for the manufacturing process of electrical rotating machines. The improvements of thermal conductivity is generally achieved by the dispersion of high thermal conductive (HTC) particles, such as BN, SiC and Al_2_O_3_ within a conventional resin [1]. However, for successful approaches in medium and high-voltage electrical insulation, it is necessary to obtain a clear understanding of the influence of particle size and shape distribution and also, the role of the interface between particles and neat resin system on the composite properties.

There are several studies on the improvements of thermal conductivity for high-voltage electrical insulation applications [1]. Lee *et al.* [45] investigated various inorganic fillers including AlN, wollastonite, SiC whisker and BN. Particles with different shape and size were used alone or in combination to prepare thermally conductive polymer composites. With respect to AlN, a titanate-coupling agent was employed for the surface treatment of the inorganic fillers. The application of hybrid fillers resulted in an increase of composites thermal conductivity, which was attributed to the connectivity enhancement offered by structuring fillers. For the same content of filler, the use of larger and surface treated particles has lead to an enhanced thermal conductivity of composite materials. On the other hand, the surface treatment of filler allowed the production of composites with lower coefficient of thermal expansion.

Zweifel *et al.* [46] investigated the potential advantages and uses of submicron and micron-sized fillers (BN, SiC and diamond) for thermal management in reinforced composites applied in electrical insulation systems. Particularly, the effect of type, size, concentration and dimensions of the fillers on the properties (electrical, thermal, mechanical, *etc.*) of reinforced epoxy laminates was determined in detail. It was also found that an improvement of thermal properties of the corresponding composite material could be accomplished whilst a minimal change in the dielectric properties was observed. Zhang *et al.* [1,47] studied the overall thermal conductivity of epoxy resin composites with the addition of selected inorganic fillers (BN, Al_2_O_3_, SiO_2_, diamond). These individual fillers have high thermal conductivity and their average size span the nano to micro dimensions. In order to obtain high thermal conductivity systems, these fillers were used singly or combined with other fillers in the epoxy resins. The results suggest that the size of BN (BN-Micro, BN-Meso and BN-Nano) is not necessary crucial to the thermal conductivity of the epoxy/hardener/filler composites at low to moderate concentrations. In terms of α-Al_2_O_3_, nano-diamond, nano β-SiC and nano amorphous silicon nitride (Si_3_N_4_) the results evidenced that the performance of these fillers is not so good as BN regarding the enhancement of the thermal conductivity of epoxy resin composites despite the fillers have comparable or in some cases higher conductivities than BN. Other studies [37,48] were performed on the thermal conductivity of different micro and nanocomposites containing selected inorganic fillers such as Al_2_O_3_, AlN and magnesium oxide (MgO). It was observed that the thermal conductivity values of the nanocomposites depend on several factors including interfacial layer structure, dimensions, and specific surface area.

Along with epoxy based thermosetting resins, thermoplastics are another class of polymer materials that are employed in electrical applications. In particular, PE was used as insulating material for medium-voltage and high-voltage energy cables in the early 1970s. After the occurring of massive cable failures due to quality problems and moisture diffusion, insulating systems of cables based on PE were replaced by XLPE. First modern XLPE cables were mainly employed for alternating current (AC) applications due to space charge accumulation purposes, but nowadays the cable technology is sophisticated enough that also high-voltage direct current (HVDC) cables can be in used in service. At the beginning of the HVDC, a general-purpose of cable development was the addition of inorganic filler into the base polymer [49]. However, in the process of adapting the cable to higher voltage application, miniaturization, higher purification and higher distribution of the inorganic filler material were pursued.

First experiments on nanocomposite insulation materials with inorganic nanoparticles distributed uniformly into the polymer matrix were performed, in order to gain advanced properties, such as improved space charge accumulation, volume resistivity, thermal conductivity, direct current (DC) breakdown strength, and lifetime in service of the insulation system. Different types of nanofiller materials were used, such as layered silicate (LS), SiO_2_, TiO_2_, and Al_2_O_3_ [50]. An example of the achieved enhancements was described by Lee *et al.* [51], where conventional AC-XLPE, DC-XLPE and nano-DC-XLPE cable insulation materials properties were compared. The volume resistivity and space charge characteristics were investigated. Between AC and DC breakdown strength a factor of more than two was demonstrated whilst the volume resistivity and the electric field loading capacity could be enhanced significantly due to the addition of nanofillers. Regarding AC XLPE cables, voltages up to 550 kV and a rated power up to 1.5 GVA were realized. The cross section of the copper conductor is up to 2500 mm² and a cable length can be 1000 m without joints. At DC XLPE cables, voltages up to 320 kV and a rated power of 1 GVA have been realized as well as cables with a length of several 100 km for subsea projects. For example an Extra High-Voltage (EHV) cable of ± 320 kV DC was applied for the interconnection between Spain and France. Underground and submarine cables have been in use since the early stages of electricity transmission [52].

High-voltage alternating current (HVAC) underground transmission cables are usually employed in densely populated areas, in submarine connections and, in general, where the implementation of overhead lines is difficult or impossible. Due to the fact that cables are installed out of sight, underground in tunnels, or under water, they have a reduced impact on the territory and a limited occupation of the soil. Terminal ends are often the only visible evidence for the presence of underground cables. The development of the power cable technology is a rather slow process. Due to the efforts of the cable industry in the recent years, a solid dielectric transmission cable with XLPE insulation is now available.

It is expected that the adoption of this type of cable will give a strong input to the realization of HVAC and HVDC underground transmission lines in the near future. Therefore, the trend towards nanodielectric research comes from the emerging need of power engineers to design new electrical insulation systems that are capable of withstanding higher voltage levels, such as HVAC and HVDC applications [53].

## 4. Polymers and (Nano)Fillers

### 4.1. Polymers Used in High-Voltage Applications

The polymer matrix, which can be incorporated into the structure of micro/nanocomposite materials used in high-voltage applications, can be divided into three major categories: thermoplastics, thermosets and elastomers. Polymers are classified in these categories as a function of their different properties, such as physical and chemical structure, thermal characteristics, mechanical and electrical behavior, *etc.*

Thermoplastic polymers are defined as plastics that become moldable above a specific temperature and solidify upon cooling. Typical examples are PE, PP, PVC, linear polyester and polyamides (PAs). Almost 85% from the global polymer production are thermoplastics and in dependence on their transition temperature characteristics, they can be divided into two large classes: amorphous and crystalline [54]. With respect to amorphous thermoplastics, such as PVC and polyamide-imide (PAI), their modulus decreases rapidly above glass transition temperature (*T*_g_), and the polymer exhibits liquid-like properties. Crystalline or semicrystalline thermoplastics, such as low-density polyethylene (LDPE), ethylene-vinyl-acetate (EVA), polyetherketone (PEK), are normally processed above the melting temperature (*T*_m_) of the crystalline phase and the *T*_g_ of the coexisting amorphous phase. Their degree of crystallinity is ranging from 20% to 90% and upon cooling, crystallization must occur quickly [54]. The large volume of low cost commodity types, such as PEs, isotactic polypropylene (i-PP), PS and PVC, represents over 70% of the total production of thermoplastics. Polymers such as acetals, PAs, PC, polyesters, polyphenylene oxide (PPO), blends and specialty polymers (liquid-crystal polymers, PEK, polyimide (PI), fluoropolymers, *etc.*) are increasingly used in high-performance applications [54].

Thermosetting polymers are pre-polymers, which form a three-dimensional polymer network upon a curing step. Curing can be accomplished either by heat (generally, above 200 °C) or by high-energy irradiation. Examples of this type of polymers are epoxy resins, polyester resins fiberglass systems, PURs, PIs, urea, *etc.* Fillers or fibrous reinforcements are often applied to enhance both properties, thermal and dimensional stability of thermosetting resins [54]. Due to their excessive brittleness, many thermosetting polymers could be useless if they are not combined with fillers and reinforcing fibers [54].

Elastomers are flexible polymers that comprise a low crosslink density and generally have low Young’s modulus and high failure strain compared to other materials. There are two main categories of elastomers: elastomer with C=C double bonds in their polymer structure (*i.e.*, styrene-butadiene copolymers, polybutadiene) and elastomers containing only saturated C–C bonds in their structure (*i.e.*, EVA, ethylene propylene diene rubber).

Among the most used polymers in micro and nanocomposites are PE (for power cables), epoxy resins, polyester, silicone and imide (for electric machines, dry transformers) and silicone rubbers (for electric insulators). PEs (low, medium or high density) hold very good electrical, mechanical and rheological properties, they are resistant to the environmental conditions, but they have low operating temperatures (below 90 °C) [11]. The addition of inorganic fillers increases the service temperature and improves their mechanical properties. Thermosetting resins have higher service temperatures (155 °C—epoxy resins, 175 °C—polyester resins, 200 °C—silicone resins, 240 °C—imide resins), but low thermal conductivity compared to that of metal parts that come in contact [11].

### 4.2. Fillers Used in Composites

Polymer composites represent a mixture of two or more components, with two or more phases, based on polymers and fillers. The fillers may have different geometries, such as fibrous, irregular flakes, spheres, acicular and plate-like in shape, cube, block, *etc.* and they are used in a reasonable large volume concentration in polymers (> 5 vol %) [54]. They can be *continuous*, such as long fibers embedded in the polymer in regular arrangements extended across the microcomposite dimensions or *discontinuous*, such as short fibers, flakes, platelets or irregularly shaped fillers (< 3 cm in length) arranged in the polymer in different and multiple geometric patterns forming a microcomposite [54].

An important diversity of fillers are in use, with different chemical compositions, shapes, forms, sizes and intrinsic properties. They are usually rigid materials, immiscible with the polymer matrix in molten or solid states forming different morphologies [54,55]. From the chemical aspects, fillers can be classified in *inorganic* (*i.e.*, oxides, hydroxides, salts, silicates, metals) and *organic* (*i.e.*, carbon, graphite, natural polymers and synthetic polymers) substances. Based on their origin, fillers can be *natural* (*i.e.*, mineral, such as asbestos and animal, such as silk, wool, cellulose, *etc.*) and *synthetic* (*i.e.*, organic fibers including Kevlar, carbon black, graphene as well as inorganic fibers such as oxides and hydroxides: TiO_2_, SiO_2_, Al_2_O_3_, aluminum trihydroxide (Al(OH)_3_), magnesium hydroxide (Mg(OH)_2_), antimony trioxide (Sb_2_O_3_), *etc.*) as summarized in Table 1 [54].

When the fillers are dispersed homogenously in the polymer matrix, in small concentrations (usually less than 10 wt %) and they are in the nanometric range, respectively with dimensions smaller than 100 nm (nanoparticles), than materials are known as nanocomposites. They are distinct from microcomposites due to their unique properties given by the interface zone formed between the polymer and nanoparticles. These interfaces are significantly increased compared to micro-sized composites, due to the nanometric scale of the particles. Because of their unique properties, nanocomposites have great potential for advanced applications.

Nanofillers can be classified in three main categories [56]:

- One-dimensional nanofiller: plates, laminas and shells,
- Two-dimensional nanofiller: nanotubes and nanofibers,
- Three-dimensional nanofiller: spherical nanoparticles.

Various nanoparticles, such as nanoclays (organomodified montmorillonite, *etc.*), nano-oxides (TiO_2_, SiO_2_, Al_2_O_3_, *etc.*), carbon nanotubes (CNTs), metallic nanoparticles (Al, Fe, Ag, and Au, *etc.*), semiconducting particles (SiC, ZnO, *etc.*) have been homogeneously dispersed in polymers, as provided in Table 2, due to the increasing demand for improvement in performances of thermoplastics and thermosetting polymer materials [56,57,58].

The properties of polymer micro/nanocomposites are affected by the nature of polymer matrix and filler, their intrinsic properties, by the size and shape of the fillers, by the dispersion of the particles into the polymer matrix, by the surface functionalization and the thickness of the filler surface treatment and also, by the interactions and adhesion between the polymer matrix and the fillers.

Owned to their very small dimensions and large specific area, polymer nanocomposites possess different physical and chemical properties compared to traditional composites. Selection of the nanoparticles for a proper application depends on the desired electrical, mechanical and thermal properties. For example [59], CNTs improve the electrical and thermal resistivity and Al_2_O_3_ is usually selected for high thermal conductivity whilst TiO_2_ nanoparticles (anastase) have photocatalytic properties. Calcium carbonate (CaCO_3_) is typically used for low costs and high number of deposits and SiC is applied for mechanical strength, hardness, corrosion and non-linear electrical behavior. In particular, ZnO is employed in composites for electric stress control due to its high thermal conductivity and non-linear electrical characteristics. Nanofillers can improve or adjust significantly different properties, such as electrical, mechanical, thermal, and optical or fire-retardancy of the materials in which they are incorporated with the condition to be homogenously dispersed. A very good dispersion of the nanofillers into the polymer matrix can be achieved by using different methods and preparation techniques among them mechanical dispersion methods, including ultrasonic vibration or special sol-gel techniques, high shear energy dispersion mixing and/or through a tailored surface modification of the nanoparticles [59,60].

### 4.3. Fillers Surface Treatment

Homogeneous dispersion of the fillers into the polymer matrix is a challenge due to the immiscibility of polymer and particles [61]. Single particles tend to agglomerate due to their interfacial tension and the properties of composites are altered. Therefore, the surface treatment of the particles is very important to achieve homogenous distribution, to avoid any cluster formation in the polymer composite and to improve the adhesion between the polymer and filler [61]. Whilst surface treatment of numerous fillers are described in literature, account has to be taken into the fact that the thickness of the modified layer plays an important role in the composites properties changes [59]. Important research regarding the influence of particles surface modification on the electrical properties of composites [62,63,64] and, especially, nanocomposites was performed [61,65,66,67].

Techniques used for controlled surface modification include: (i) *chemical treatment* of the nanoparticles surface; (ii) *grafting* of functional polymeric molecules to the hydroxyl groups existing on the particles surface and (iii) *plasma* techniques, which make the surface of the particles more or less wettable, harder, rougher and more conducive to adhesion to the polymer [61].

**Figure 5:**
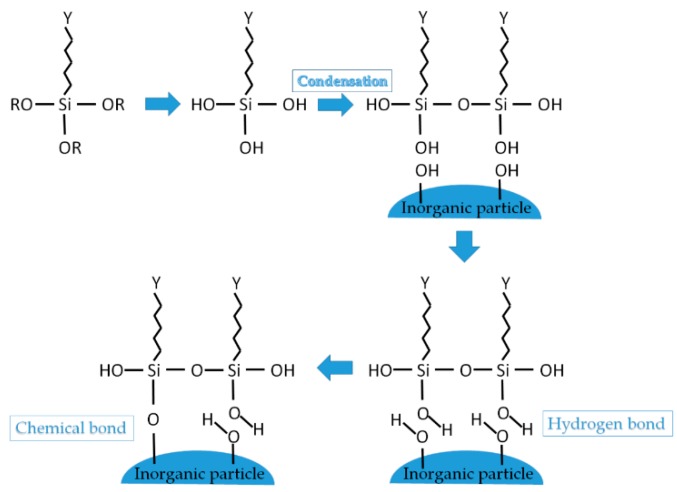
Schematic representation the surface functionalization of inorganic particles via condensation reaction of functional silanes (Redraw and adapted figure from [69]).

Usually, for the *chemical treatment* of the particle surfaces, different types of silane coupling agents (*i.e.*, 3-aminopropyl triethoxysilane) are used (Figure 5). Silane coupling agents can react with the hydroxyl groups of inorganic and organic surfaces via condensation reaction [61,68]. The change in the surface polarity in conjunction with steric hindrance effects enables a better dispersion between the modified nanoparticles and polymer matrix [61]. Another typical coupling mechanism, similar to the one described before is titanate coupling agent (*i.e.*, tetra-isopropyl titanate), where the same effect is obtained [61].

**Figure 6:**
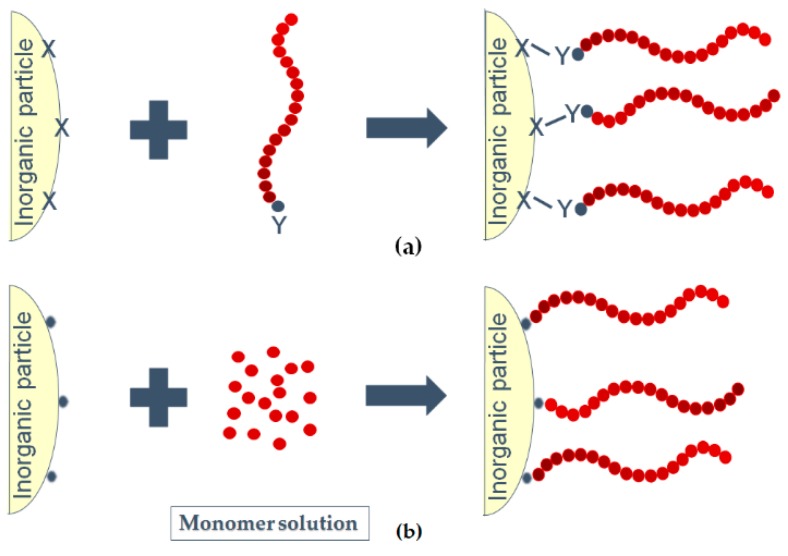
Schematic representation the surface functionalization of inorganic particles by (**a**) “grafting onto” and (**b**) “grafting from” reactions (Redraw and adapted figure from [72]).

*Grafting* of the functional polymeric molecules to the hydroxyl groups of the surface of nanoparticles is another technique to overcome the incompatibility between inorganic fillers and polymer matrix [70]. As provided in Figure 6, there are two different methods to prepare grafted surfaces. One the one hand side a polymer chain is directly coupled to the inorganic filler surface (“grafting onto” reactions) and on the other hand side the attachment of monomers on the surface and the subsequent polymerization of the polymeric chain (“grafting from” reactions) is accomplished [71]. Through this method, selected polymer chains are grafted to the nanoparticle surface by strong covalent bonding. Due to the covalent attachment of the polymer, the modified nanoparticles become either hydrophobic or hydrophilic and their miscibility is enhanced. Further separation of the nanoparticles partially results from the steric hindrance effect [61].

*Plasma* method is another technique that modifies chemically and physically the surface of the inorganic nanoparticles, without influencing the bulk properties of the fillers. In the presence of selected monomers, graft copolymerization and polymerization reactions can be carried out during plasma treatment enabling the preparation of particles with controlled surface properties. The limitation of this technique is that the experimental conditions require a very complicated vacuum system [61]. Surface modification of the inorganic particles trough these methods produces excellent integration and good adhesion between the polymer matrix and fillers [61].

### 4.4. The Role of the Interface

**Figure 7:**
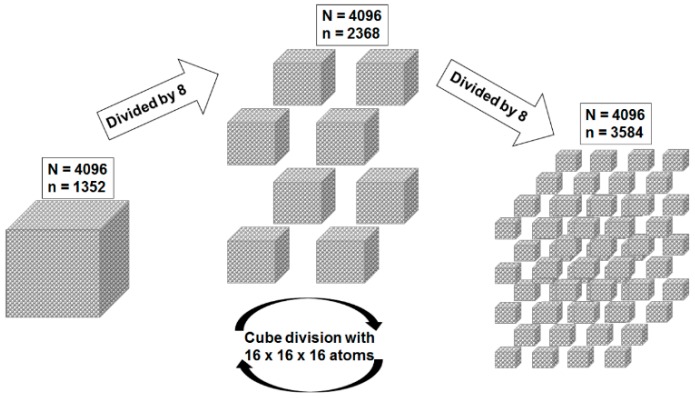
Surface statistic consequence of dividing a cube, where *N* is the total number of atoms and *n* is the number of surface atoms (Redraw and adapted figure from [73]).

Interface regions formed between the polymer matrix and particles are considered to have a dominant role in the final properties of nanocomposites [23]. Some simple examples can clearly demonstrate the significance of the interface area, especially, at the nanometric and molecular levels [69]. Taking into consideration the example of Kickelbick [73], a cube composed by 16 × 16 × 16 atoms packed tight is illustrated in Figure 7.

The cube contains a number of 4096 atoms, from which 1352 are located on the surface, therefore ~33% of the atoms are surface atoms. In the case that the cube is divided into eight equal 8 × 8 × 8 cubes, the overall number is the same, but 2368 atoms are located at the surface, which means ~58% of the atoms are located on the surface. If the same operation is repeated, 3584 atoms are obtained at the surface, which means ~88% surface atoms.

The example shows how important the surface becomes when objects are becoming smaller and smaller in size. In terms of nanoparticles, which sizes are in the range of tens nanometers, nearly every atom is a surface atom that can interact with the polymer and therefore inner surface has a direct impact on the nanocomposite properties [73].

For a better understanding of the interface properties and its physico-chemical structure, several papers were published and focused on the models development in order to describe the interface between nanoparticles and polymer matrix. All the established models started from the fundamental theories and models of dielectric materials. When composite dielectric materials are manufactured with different shapes, sizes, dielectric properties and concentration of each component, it is beneficial to predict through analytical and numerical models, the effective permittivity and the distribution of electric fields within the composite [74].

The first analytical models for estimation the effective permittivity of dielectrics were based on the effective medium theory (EMT), where the interface between the components was not considered [75]. These models assumed an elementary cell containing a spherical inclusion of an arbitrary radius embedded in a matrix, such as Maxwell–Garnett (MG) model [75]. Later, MG model was extended with different assumptions to consider the interactions between the different constituents, such as the symmetric Bruggeman model [75].

More recently analytical models were developed and among them exists the three-dimensional electromagnetic model developed by O’Connor *et al.* [74]. A software constructs a composite with thousands of distinct elements arranged according to the defined input parameters. The software provides the modeling composite systems without creating manually many individual composite elements and enables user specification over the simulation parameters, analyzing the results through a user interface. The permittivity of each composite element, concentration, shape, size, and density, are user-defined. The effective permittivity of the composite is determined by a capacitance of a plate capacitor containing the virtual composite. Comparisons between the simulated effective permittivity and the values calculated using classical equations for the effective permittivity of composites with various particle concentrations are presented. Finally, examples of how the electric field is distributed through the composite structure are included [74].

The above analytical and numerical models for predicting the effective permittivity of composite dielectric materials, do not take into account explicitly the electric charge distribution in composites, so this aspect could be one important limitation regarding the use of these models for polymer nanocomposites, in defining their dielectric properties [75].

#### 4.4.1. Wilkes’ Model

**Figure 8:**
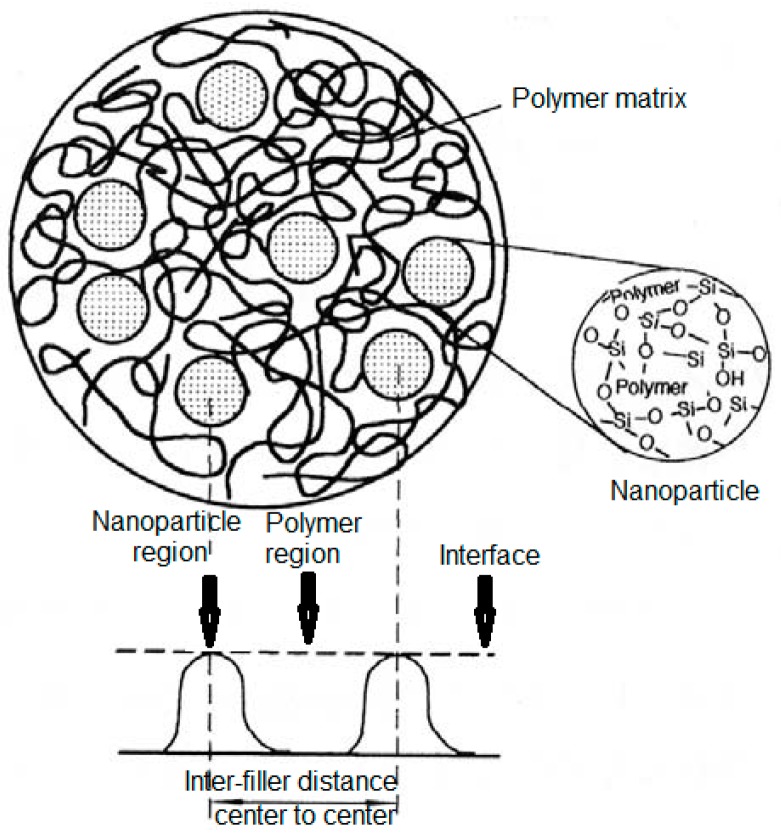
Wilkes’ model of the interface formed between silica nanoparticles and polymer matrix (© 2016 IEEE. Reprinted, with permission, from [76]).

Wilkes published the first article with an interface model in 1989 [76]. The model describes the partially distribution of the silica nanoparticles into the outer polymer region formed by covalent bonds, which were generated by the end cap method, as depicted in Figure 8 [76]. Silica nanoparticles and the polymer matrix are combined to form a network structure of hydrogen bonds produced by the direct mixing methods [23].

#### 4.4.2. Tsagaropoulos’ Model

**Figure 9:**
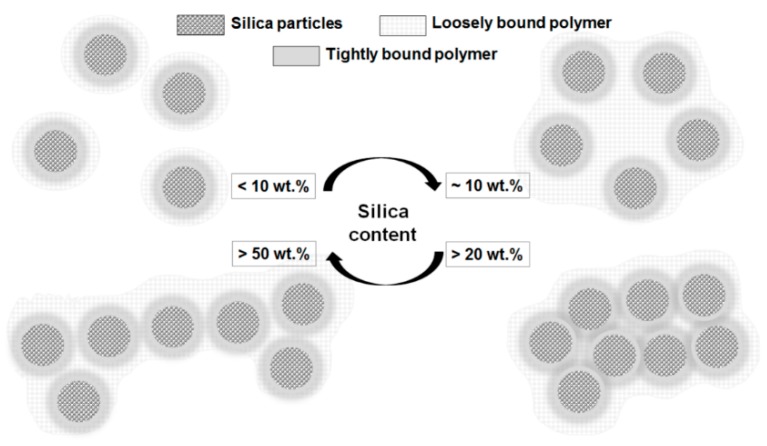
Tsagaropoulos’ schematic representation model of the morphological changes in the polymer matrix filled with silica filler in different concentrations: (**a**) less than 10 wt %; (**b**) circa 10 wt %; (**c**) over 20 wt % and (**d**) over 50 wt % (Redraw and adapted figure from [77]).

Another interface model is the one proposed by Tsagaropoulos *et al.* in Reference [77]. For a better understanding of the morphological and structural changes generated by the increase of the filler concentration within the polymer matrix, which results in a decrease of the average interparticle distance, Tsagaropoulos proposed a model represented schematically in Figure 9 [77].

The incorporation of the nanoparticles into a polymer matrix creates interactions between the nanofillers and the polymer chains located in their vicinity, generating regions with restricted chain mobility around nanoparticles [78]. These restricted mobility regions possess their own glass transition temperature (*T*_g_), according to the model for the morphology of ransom ionomers (EHM) and the model of Tsagaropoulos is focused on *T*_g_ behavior in different combinations of polymers and silica nanocomposites [77,78].

Tsagaropoulos’ model assumes that silica particles (textured areas in Figure 9) are surrounded by a layer of polymer (grey areas in Figure 9) or tightly bound layer, which appears to be immobile in the temperature and frequency regimes and does not participate to the glass transition. The polymer chains capable to participate to the glass transition (textured light grey area in Figure 9) are called polymer of reduced mobility or loosely bound layer [77]. A series of experiments were accomplished on different polymer/silica nanocomposites and they revealed the existence of two glass transitions. One is the glass transition of the polymer and the other is the glass transition of the loosely bound layer. This conclusion came from the experimental appears of two tan δ peaks [77,78].

#### 4.4.3. Lewis’ Model

**Figure 10:**
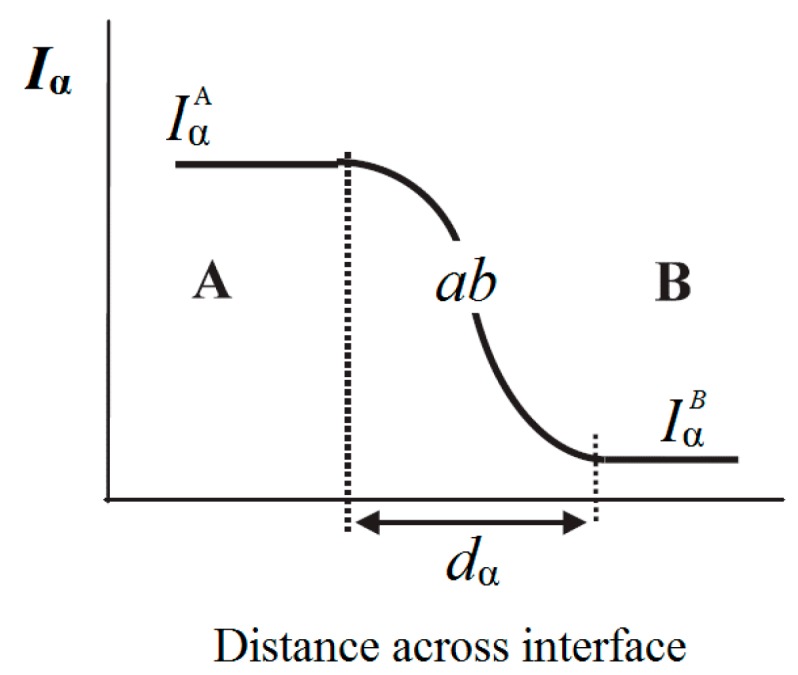
Intensity model of Lewis showing the interface *ab* between two phases A and B are defined by the intensity *I*_α_ and the changes of the property α suffered when crosses the interface (© IOP Publishing. Reproduced with permission. All rights reserved [79]).

In 1994, Lewis highlighted the importance of the interface considering them as regions with altered electrochemical and electromechanical behavior [16]. In 2004, Lewis defined an interface between two uniform material phases A and B, as illustrated in Figure 10 [79]. The intensity *I*_α_ of a chosen material property α associated with the forces is constant within each of the two phases A or B, but will become increasingly modified as the interface with another phase is approached [79,80]. In general, α can be any physical or chemical property (*i.e.*, electrochemical potential, the electric field, the local dielectric permittivity or an optical parameter) [79].

**Figure 11:**
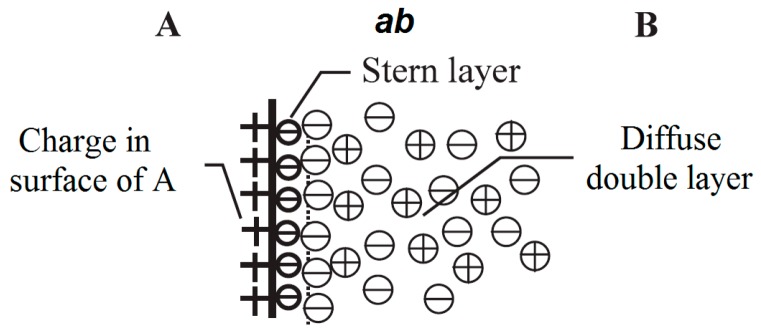
Distribution of the electrical potential in Stern layer and Gouy-Chapman diffuse mobile ion double layer of the interface *ab* in response to a charge A (© IOP Publishing. Reproduced with permission. All rights reserved [79]).

The region over which the forces associated to a chosen property α is different from the bulk values in each phase is called interface *ab* where the intensity *d*_α_ changes from $I_{\alpha }^{A}$ in the bulk phase A to $I_{\alpha }^{B}$ in the bulk phase B [79,80]. Lewis also suggested that an electrical charge layer is formed around the nanoparticles in the interface region *ab*, as shown in Figure 11 [79].

The interface *ab* consists of three charged layers (see Figure 11). On the side A of the interface *ab*, there is a double layer associated with the surface of phase A, which is formed by trapped carriers, mobile electrons and holes and immobile charged impurities [79]. Next to the first layer, a double layer with a higher charge density, termed Stern (Helmholtz) layer), exists. The A side of the Stern (Helmholtz) layer is formed by absorbed ions and dipoles, whilst its B side is determined by ions attracted by the excess of charge on phase A. Beyond the Stern (Helmholtz) layer there is the Gouy-Chapman layer, which is formed by the separation of mobile positive and negative charges from phase B. The interface *ab* charging involves electronic polarization and orientation of any permanent dipole and is described as Stern/Gouy-Chapman double layer model. The electrical double layer is composed by the compact Stern layer, formed by charges immediately adjacent to the surface A and Gouy-Chapman layer, which is the more distant diffuse part [79,80]. If medium B has a polar component, then the charge is significant. If medium B contains mobile ions, they will immigrate to establish a diffuse electric double layer around particle A [80].

#### 4.4.4. Tanaka’s Model

**Figure 12:**
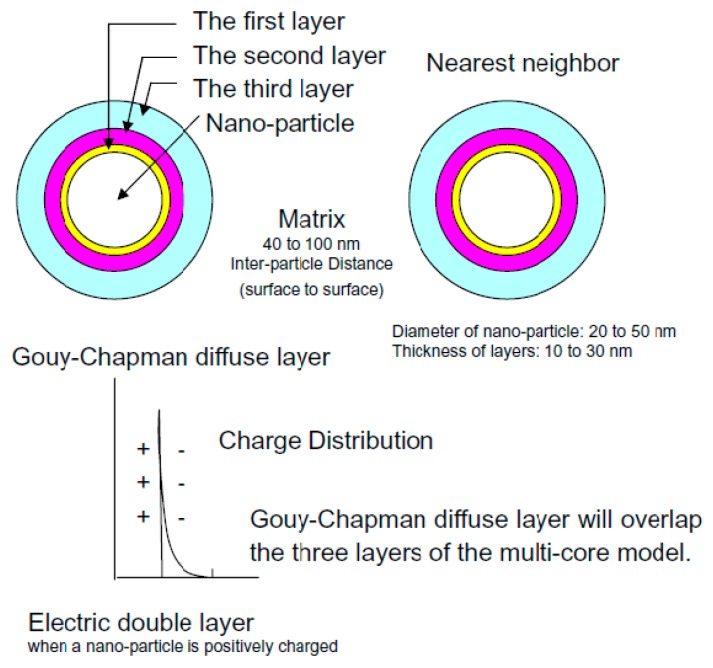
Tanaka’s multi-core model (© 2016 IEEE. Reprinted, with permission, from [76]).

In 2005, Tanaka proposed a multi-core model in one of his paper [76] in order to understand better various properties and phenomena that polymer nanocomposites exhibit as dielectrics and electrical insulation. This model describes the physico-chemical and electrical structure of the interface regions formed between spherical nanoparticles and polymer matrix (see Figure 12) [76].

According to Tanaka’s model, the interface is defined as a multi-layer of several tens nm and is formed by three layers: a bonded layer (the first layer), a bound layer (the second layer) and a loose layer (the third layer). A Gouy-Chapman diffuse layer is superimposed on the three layers of the interface and cause a far-field effect [76]. The first layer is a region of chemical bonding between the inorganic particles and organic polymer matrix. The second layer is an interfacial region consisting of a layer of polymer chains strongly bound and/or interacted with the first layer and the surface of inorganic nanoparticles. The third layer is a region loosely coupling and interacting with the second layer, with a different morphology compared to the others [76].

In polymer nanocomposites, the particles may interact electrically with the nearest neighbors’ particles due to the far-field effect, resulting in a collaborative effect [76]. This model can explain different electrical properties, such as partial discharges resistance of PA/LS nanocomposites [76]. Some articles were published in order to show Lewis’ contribution in the developments of Tsagaropoulos’ and Tanaka’s models, their differences and similarities and future challenges [81,82].

#### 4.4.5. Other Models

Besides these four models of the interface region between nanoparticles and polymer matrix, various models were proposed, but the matching between models and experimental results is quantitatively poor. Computer simulation and numerical modeling are expected to generate more quantitative results.

**Figure 13:**
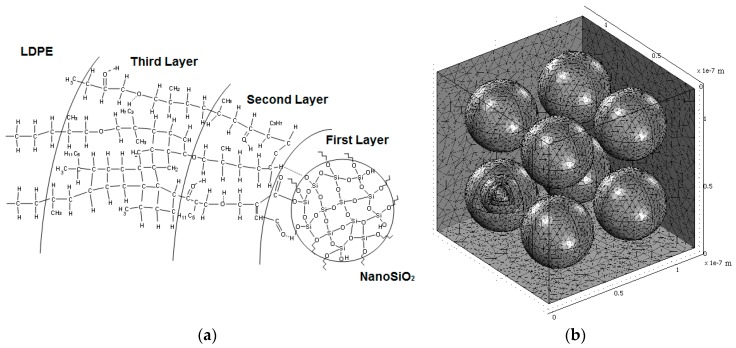
(**a**) Schematic representation of an interface LDPE—nanoSiO_2_ chemical structure and (**b**) 3D electrostatic model (Reprinted, with permission, from author [84]).

Starting from Tanaka’s model, in 2008 an electrostatic 3D model by Ciuprina *et al.* [75] was proposed in order to analyze the electric field distribution inside and outside of the spherical nanoparticles homogenously dispersed into a polymer matrix. In addition, this model can reveal the impact of the nanoparticles diameter and concentration, the thickness of the interface layers and the permittivity of the nanoparticles and layers on the polymer nanocomposites properties [83]. Starting from similar hypothesis, in 2012, Plesa [84] proposed a novel structural model of nanocomposites composed of LDPE filled with inorganic nanoparticles (SiO_2_/TiO_2_/Al_2_O_3_) (Figure 13) [84]. The thicknesses of the LDPE-nanofillers interfaces were assumed to differ in size. Types of electric dipoles present on the interfaces were identified depending on the chemical structures of the individual layers, and a calculation was done to estimate their concentration. The permittivity values of the interface layers were computed with the proposed model assuming no interaction between the dipoles. The achieved data were compared with the experimental results obtained on the same type of nanocomposites and they were used to explain the interface structure and the electrical properties of nanocomposites [84].

**Figure 14:**
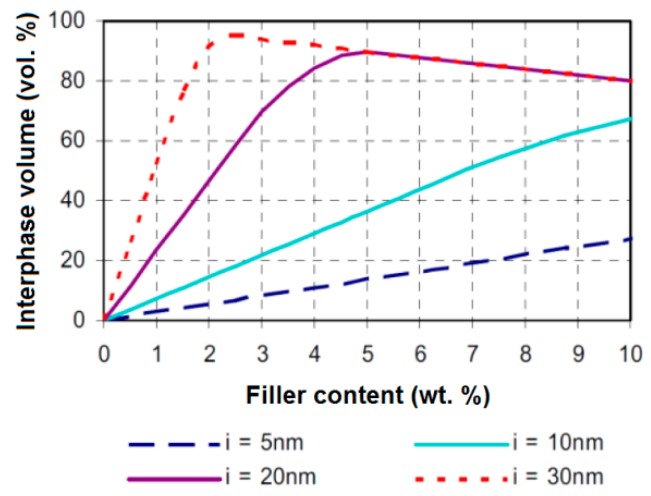
Interphase volume model of Raetzke for a silicone matrix and SiO_2_ particles, with different interface thicknesses *i* (© 2016 IEEE. Reprinted, with permission, from [85]).

Another model influenced by the multi-core model of Tanaka is the interphase volume model proposed by Raetzke in 2006. According to this model [80,85], the term interface is replaced by the term interphase of which characteristics depend on the particles size, filler concentration and type of polymers and nanofillers. Within the hypothesis of an ideally nanoparticles dispersion in a polymer matrix, a certain interphase thickness is assumed and a maximum of interphase volume is reached for a distinct filling concentration (see Figure 14) [80,85].

Comparing the dependence of the interphase volume on the fillers concentrations with the dependence of the electrical properties of the polymer nanocomposites on the filler content, the interphase thickness within the nanodielectrics can be estimated [80,85].

**Figure 15:**
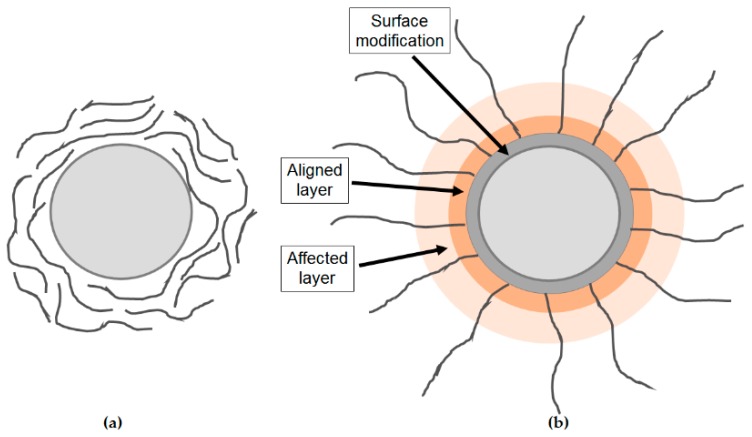
Polymer Chain Alignment Model by Andritsch: nanoparticles (**a**) without and (**b**) with surface modifications. (Redraw and adapted figure from [86]).

The polymer chain alignment model proposed by Andritsch in 2011 [80,86] is based on experiments and describes the morphology of nanofilled epoxy resins (Figure 15).

If the nanoparticles are unmodified (Figure 15a), the interactions between the nanoparticles and the polymer matrix are low. If the surfaces of nanoparticles are modified with a silane coupling agent (Figure 15b), a restructuring of the polymer matrix will take place due to the reactions between the polymer and the silane epoxy groups: an alignment layer of the polymer chains perpendicular to the nanoparticle surface appears and the polymer surrounding region is affected, too [80,86].

**Figure 16:**
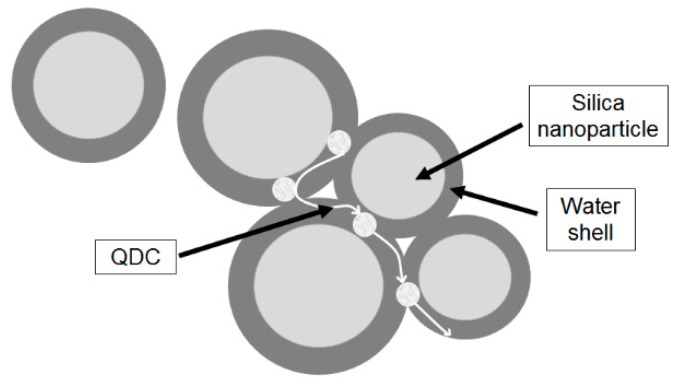
Schematic representation of the water shell model proposed by Zou. The percolative path passes through overlapping water shells, around nanoparticles (Redraw and adapted figure from [87]).

The water shell model proposed by Zou *et al.* in 2008 [87] is based on Lewis’ and Tanaka’s models and explains the effect of water absorption in epoxy nanocomposites, when they are exposed to humidity [78]. In this model is considered that the water molecules are concentrated around the nanoparticles and, in low concentrations, in the polymer matrix. If the water concentration around the nanoparticles is high, percolative paths are formed through overlapping water shells (Figure 16), which affect the dielectric properties of epoxy nanocomposites [78,87].

The models of polymer nanocomposites presented above give an idea about the physico-chemical and electrical properties of the interface between nanoparticles and polymer matrix. Parts of the experimental results were explained through these models, but with some limitation since the interface regions have not been made visible until now in polymer nanocomposites.

## 5. Preparation Methods

Preparation methods of microcomposites are relatively simple and these can be manufactured in large quantities [54]. Taking into consideration different types of polymer and fibers from which polymer microcomposites can be made, the preparation methods represent a broad subject [88]. The basic steps include impregnation of the fiber with the resin, forming of the structure, curing of thermosets or thermal processing of thermoplastic matrices and finishing [88].

Depending on the process, these steps may occur separately or continuously. For example, the starting material for many polymer microcomposites is a prepreg process, where fiber tapes or cloths that have been preimpregnated with resin, are partially cured. Pultrusion, impregnation, forming and curing are done in a continuous process. Some other important manufacturing techniques are: sheet molding (fast flexible technique), injection molding (fast technique, high volume of fibers in thermoplastics matrices), resin transfer molding (fast technique, complex parts and good control of fiber orientation), prepreg tape lay-up (slow and laborious technique, reliable, expensive), pultrusion (continuous technique, constant cross-section parts), filament winding (moderate speed technique, complex geometries and hollow parts) and thermal forming (fast, easy, repair and joining technique, reinforced thermoplastics) [88].

Over the last two decades, chemists and material scientists have shown significant interest and important development on the preparation methods of organic and/or inorganic nanocomposites [89]. Nanocomposite materials can be obtained using similar microcomposites design and synthesis techniques, which make them interesting from the production point of view [90]. The incorporation of nanoparticles into the polymer matrix can lead to novel high-performance compared to the unfilled or micro-filled polymers [89].

In order to obtain these high-performances in thermal, mechanical or electrical properties, nanofillers should be homogeneously dispersed into the polymer matrix and should be physically or chemically bounded by the surrounding polymer [89]. During the fabrication process, agglomerations of nanoparticles tend to appear due to the interfacial tension, accumulated on the nanofillers surface and due to the incompatibility of inorganic and organic components. It was recognized that the surface treatment of nanoparticles would bring a better dispersion in the polymer matrix [89]. The correctly chosen curing dispersion agent will bind the organic polymers and inorganic particles, which are immiscible [89]. Taking into consideration all these aspects, a certain number of techniques were developed in order to obtain improved nanocomposite materials.

**Figure 17:**
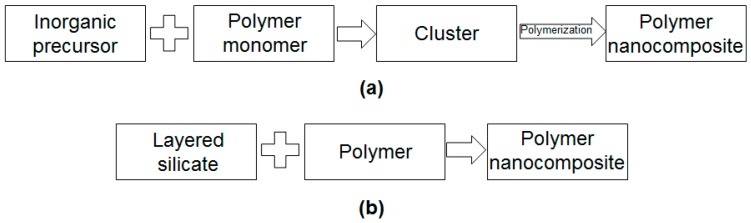
Schematic representation of (**a**) bottom-up and (**b**) top-down methods.

Usually, the synthesis of polymers nanocomposites applies bottom-up or top-down methods [90]. Bottom-up methods involves chemical processes (sol-gel process, chemical vapor deposition, spray pyrolysis, *etc.*), where the precursors are used to construct and grow organized structures, from the nanometric level (see Figure 17a). Top-down techniques top-down are using in the most cases physical methods (LSs dispersion in polymer, *etc.*) and the bulk material is broken down into smaller pieces or patterning (see Figure 17b) [90].

Inorganic nanofillers can be dispersed into the polymer matrices via four different ways: (i) *intercalation method* based on the exfoliation of LSs; (ii) *sol-gel process* which starts, at room temperature, with a molecular precursor and then forms by hydrolysis and condensation reactions a metal oxide framework; (iii) *in situ* formation of nanofillers and *in situ* polymerization of monomers in the presence of fillers previously obtained and (iv *direct mixing* of the polymer and the nanofillers, either as discrete phases (melt mixing), or in solution (solution mixing) [91]. In the following section, well-established preparation techniques to obtain nanocomposites materials are described.

### 5.1. Intercalation Method

Intercalation method is a typical top-down method based on the decreasing size of filler to the nanometric scale [92]. This method can be achieved by three ways: direct intercalation of polymer chains from solution, polymer melt intercalation and intercalation of monomers followed by *in situ* polymerization [73].

#### 5.1.1. Direct Intercalation of Polymer Chains from Solution

**Figure 18:**
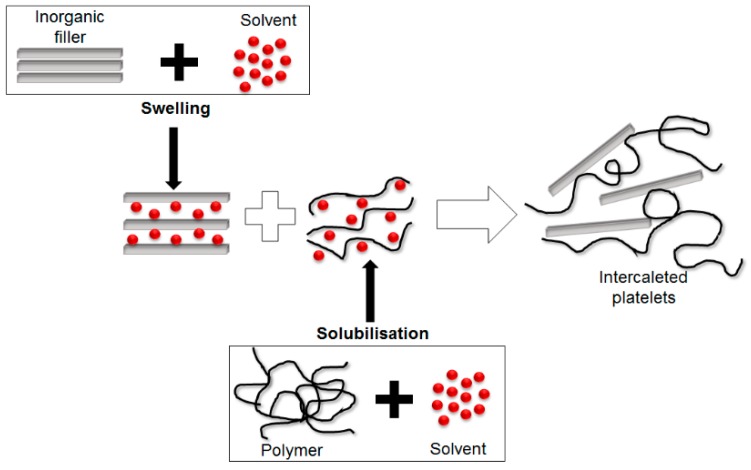
Schematic representation of the solution processing (Redraw and adapted figure from [93]).

Direct intercalation of polymer chains from solution is the procedure of dispersing layered fillers (*i.e.*, silicates) into a solvent in which the polymer is soluble and is known as exfoliation or adsorption process. When the solvent is eliminated from the polymer-clay complex through evaporation, the silicates sandwich the polymer to form a multi-layered structure (see Figure 18) [91,93].

#### 5.1.2. Polymer Melt Intercalation

**Figure 19:**
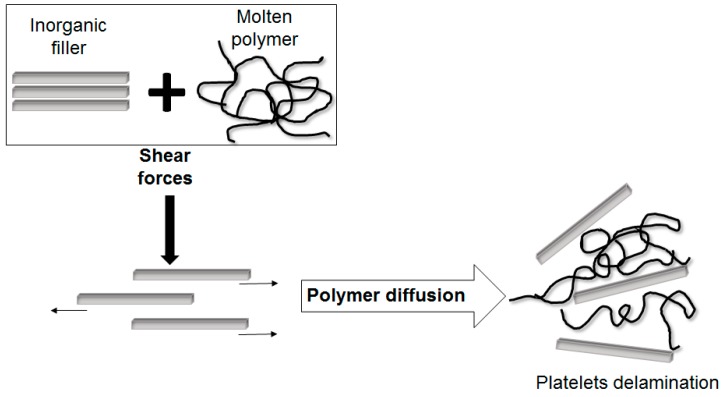
Schematic representation of the melt processing (Redraw and adapted figure from [93]).

Polymer melt intercalation involves the mixing of the layered filler (*i.e.*, silicate) with the polymer in the molten state. If the surfaces of the silicate layers are sufficiently compatible with the polymer chains, the polymer can be inserted into the interlayer space, without any solvent, forming intercalated or exfoliated nanocomposites (see Figure 19) [91,93].

#### 5.1.3. Intercalation of Monomers Followed by *In Situ* Polymerization

**Figure 20:**
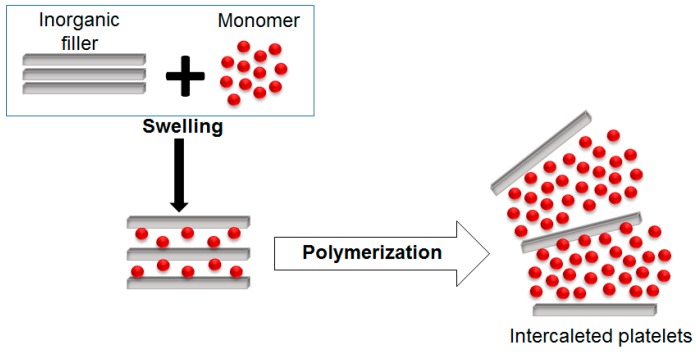
Schematic representation of the intercalation of monomers *in-situ* polymerization (Redraw and adapted figure from [93]).

Intercalation of monomers followed by *in situ* polymerization is the procedure that uses monomers with initiators, which are allowed to polymerize in the presence of the layered filler (one prominent example is clay). During the polymer chains growing, the clay layers are separated and the polymer chains enter the interlayer space, forming polymer/clay nanocomposites (see Figure 20) [91,93].

### 5.2. Sol-Gel Method

Sol-gel method is a typical bottom-up method and is associated with two reactions steps, namely sol and gel. Sol represents a colloidal suspension of solid particles in a liquid phase and gel is the interconnected network formed between phases [90]. This process consists of two main reactions: hydrolysis of the metal alkoxides (Equation (1)) and condensation of the hydrolyzed intermediates (Equations (2) and (2′)) [90]:
•Hydrolysis:M(OR)_4_ + H_2_O → HO − M(OR)_3_ + ROH,(1)•Condensation:(OR)_3_M − OH + OH − M(OR)_3_ → (OR)_3_M − O − M(OR)_3_ + H_2_O,(2)(OR)_3_M − OH + RO − M(OR)_3_ → (OR)_3_M − O − M(OR)_3_ + ROH.(2’)

Both are multi-steps processes and occur sequentially [90]. This method can be used in order to obtain inorganic metal oxides from organic metal alkoxides, esters, *etc.* and transparent films of organic-inorganic hybrid trough co-hydrolysis and polycondensation of alkyltrimethoxysilane-tetramethoxysilane mixtures [91]. The sol-gel procedure is a useful way to produce inorganic-organic hybrids [91].

### 5.3. In Situ Polymerization

Formation of nanoparticles via *in situ* polymerization is a method of synthetisation of nanoparticles via polymerization of colloidal sols containing metal ions and monomers. The size of the nanoparticles depends on the experimental conditions (temperature, thermal coagulation, *etc.*) and colloidal sols properties [91]. This method is used to prepare nanocomposites based on thermosetting polymers and nanoparticles, which are dispersed within monomer (or monomer solution) and the mixtures-polymerized by standard methods [92].

### 5.4. Direct Mixing of Nanoparticles with the Polymer

**Figure 21:**
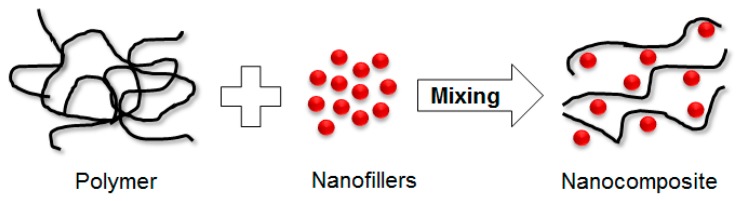
Schematic representation of the mechanical mixing of nanoparticles with the polymer.

Direct mixing of nanoparticles with the polymer is a typical top-down approach and is the simplest method to obtain nanocomposites. This method involves, either direct mechanical mixing of the polymer with nanofillers (in the absence of any solvent), above the softening point of the polymer (termed melt-compounding method) or mixing the polymer and fillers as a solution (termed solution-mixing method) [23,92]. Due to the surface treatment of the nanoparticles and the development of mixing equipment, homogenous samples can be obtained through this method (see Figure 21).

Polymer nanocomposites represent very promising materials for applications as dielectrics and electrical insulations, from the viewpoint of their excellent properties [33]. Nevertheless, in order to move these materials from the lab to an industrial production, it is necessary to develop suitable methods towards the large-scale manufacturing of polymer nanocomposites. This will help to create reproducible and reliable data that are needed for expanding development of these advanced materials in the high-voltage applications.

## 6. Properties of (Nano)Composites

It is a challenge to design and optimize proper electrical insulation systems of electrical equipment, in the conditions when energy demands, voltage level and temperature values are increasing and on the other hand, the electrical components and equipment sizes are becoming smaller and more compact compared to the traditional ones, increasing the demands on the insulation systems. Thus, current research aims at systems that are expected to have better endurance and reliability compared to their conventional counterparts [94].

In the insulation engineering, polymer composites are the second generation of what is called filled resins and consists of polymers filled with a large amount (the order of 50 wt %) of inorganic microfillers [33]. They are traditionally designed to be used as structural materials [95]. Due to the rapid growth of the electrical engineering industry, composite materials were involved more and more in electrical applications, as structural and electronic composites. However, there is a vast difference in their property requirements: while structural composites emphasis high strength and high modulus, electronic composites emphasis high thermal conductivity and low thermal expansion. Considering all these aspects, the industry is continuously looking for better alternative materials, which come at affordable prices to maintain the requisite price margin, besides considering the depleting natural resources of conventional materials [95].

Composite materials offers some advantages in terms of light-weight, resistance, ease of maintenance and better environmental protections, but also poses some disadvantages in terms of processing costs and the choice of materials. Composite materials offers the opportunity to provide the suitable product with required performance for the final application and thereby optimizing the price-performance ratio [95]. In high-voltage applications, solid electrical insulation materials, termed dielectrics, in the early days of electrical power applications were made of natural materials and ceramic materials [94,96]. There were two areas where few advances had been observed for cellulose-based paper, which is still the main insulation system in power transformers and sub-water cables applications and outdoor electric insulation materials for high-voltage lines and bushing [94]. However, polymer composites with better performance as well as lower weight than the conventional polymers were developed in the last several decades. Later, the use of plastics and epoxy resins in electrical engineering applications, allowed the manufacturing of insulation systems with controlled properties. In general, plastics are easier to be shape and process compared to glass and ceramics, but do not possess sufficient mechanical strength. Light-weight designs of polymer composites (containing inorganic fillers) with enhanced material properties were pursued in the last several decades. Epoxies offered new possibilities in developing electrical insulation, particularly to reach a more compact design in electrical power equipment [97]. Fillers have been introduced to improve the mechanical and other properties (electrical, thermal) of polymers [94]. Due to their good adaptability and simple manufacturing technology, mineral filled epoxies are the preferred materials for indoor and outdoor insulation. The disadvantage of these materials is long-term aging [97]. Nowadays, polymeric epoxy resins, hardeners and industrially available fillers are of good quality, but their internal interfaces, which lead to aging, cannot be avoided [97].

Newly born emerging advanced materials, called polymer nanocomposites are defined as polymers filled with a small amount of nanofillers (few wt % in content and 1 to 100 nm in size), which should be homogeneously dispersed in the polymer matrix and poses a tremendously large surface as compared to microfillers [33]. Therefore, in order to understand the characteristics that emerge due to nanostructuration, it is indispensable to investigate the interaction between nanofillers and polymer matrix [33]. These materials possess a huge potential in applications, such as building, transportation, food packaging, electrical and electronics engineering, industries, *etc.* and could be used as high functional materials (coating and barrier-functional materials, flame-retardant and foamed materials, *etc.*) [33].

First experimental results on the electrical properties of polymer nanocomposites were reported by Nelson and Fothergill in 2002 [98]. Their investigations on epoxy systems filled with TiO_2_ micro/nanoparticles concluded that: (i) nanofilled epoxy exhibits a flat loss tangent response at low frequency compared to microcomposites; (ii) nanofilled epoxy exhibits mitigated space charge behavior compared to microcomposites and (iii) the decay of charge for nanofilled epoxy is rapid compared with microfilled epoxy [53,98]. Whereas conventional microfilled materials reduce dielectric strength due to bulk charge accumulation, they reported [22] that the nanofilled epoxy exhibited a higher DC breakdown strength compared to microcomposites, but the values were close to the base polymer. Subsequent experimental findings on the use of nanocomposites in electrical insulation were overwhelming positive [53].

Various nanocomposite systems, such as PE/TiO_2_, PP/LS, EVA/LS, epoxy/TiO_2_, epoxy/Al_2_O_3_, epoxy/ZnO, were investigated and it was reported that space charge formation was mitigated upon nanostructuration and showed reduced charge accumulation at high fields when compared with the base polymer [53]. Meanwhile, the breakdown performance of various nanocomposite systems was found to be enhanced compared to equivalent microcomposites [53]. In addition, nanocomposites were generally more resistive to partial discharges compared to microcomposites and base polymers. Because microfillers are much less closely packed as nanofillers, erosion of the matrix around the nanofillers was assumed to proceed as in the unfilled polymer. Similar mechanisms were also suggested for tree-retardant effect found in nanocomposites [53]. Nanocomposites were found to exhibit lower permittivity and loss tangent compared to microcomposites and sometimes to unfilled polymer [53]. Mechanical and thermal properties, such as tensile strength, bending strength, elastic modulus, weight deflection temperature and heat decomposition temperature were found to be improved for various types of nanocomposites [33]. Long-term characteristics such as creep, stress relaxation and fatigue were obtained for different nanocomposites systems. Furthermore, some types of nanocomposites exhibit characteristics that are particularly important for selected target applications, such as paint performance, high biodegradability, gas barrier effect, flame retardancy, foaming ability, *etc.* [33].

Nanodielectrics are expected to possess unique dielectric properties due to the interfacial region between nanofillers and polymer, rather than a simple binary combination of properties, such as in conventional microcomposites. This distinct property lead to the idea of a new class of dielectric materials with combined electrical, mechanical and thermal properties and can be an excellent class of material as far as AC and DC applications are concerned [53]. Nevertheless, many promising experimental results have been reported concerning the use of nanodielectrics as electrical insulation materials in high-voltage applications.

### 6.1. Electrical Properties

#### 6.1.1. Electrical Conductivity

**Figure 22:**
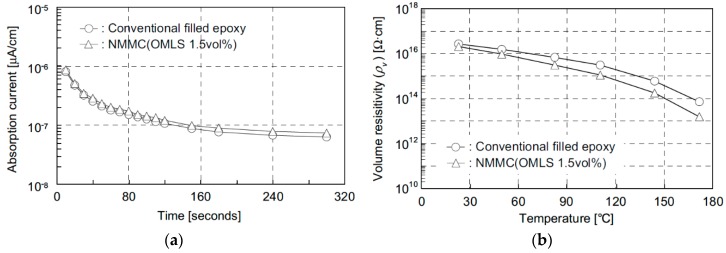
(**a**) Absorption currents in time after applying DC voltage (500 V) and (**b**) volume resistivity dependence with temperature (© 2016 IEEE. Reprinted, with permission, from [99]).

Imai *et al.* [99] investigated the electrical properties of micro/nanocomposites based on epoxy resin and LS/silica fillers. Figure 22a shows the relationship between absorption current and time.

It was found that the damping curve of the nano- and micro- filler mixture composite (NMMC) with 1.5 vol % organically modified layered silicate (OMLS) is almost the same as that of the conventionally filled epoxy and a very small influence of modifier ions of LSs is observed on these results. At room temperature there was no significant difference between the results for conventional filled epoxy resin and the NMMC/1.5 vol % OMLS on volume resistivity values, but increasing the temperature, resistivity decrease slightly in the case of nanocomposites [99].

Castellon *et al.* [26] observed that the conduction currents are significantly influenced by the SiO_2_ concentration compared to unfilled epoxy resin. The greater the concentrations of micro- and nanoparticles in the base polymer are, the greater the conduction currents are obtained [26]. All the obtained results were explained through the Schottky model approach.

Singha *et al.* [32] analyzed the variations of DC volume resistivity with respect to filler concentrations (TiO_2_, Al_2_O_3_ and ZnO) in epoxy nanocomposites. Even the introduction in the systems of free ions by adding inorganic particles, which can increase the DC conductivity of composites, their influence was not found to be significant in this study [32]. Patel *et al.* [100] performed similar studies on nanocomposites based on epoxy resin and Al_2_O_3_ nanofillers.

Lutz *et al.* [101] analyzed the influence of water absorption on volume resistivity of epoxy resin insulators. They proposed a model (based on Fickian diffusion) to simulate the dynamic process of volume resistivity decrease during humidity storage.

**Figure 23:**
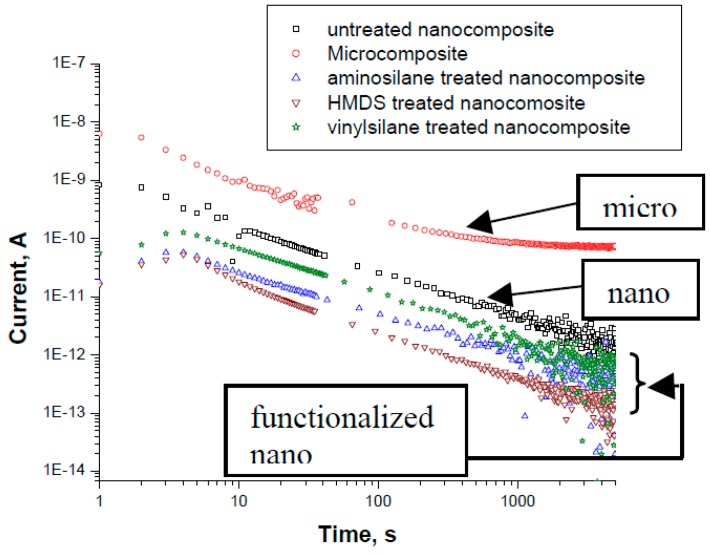
Absorption current dependent on time for XLPE/SiO_2_ micro/nanocomposites (© 2016 IEEE. Reprinted, with permission, from [102]).

Roy *et al.* [102] studied the time dependent conduction characteristics of micro/nanocomposites based on XLPE and 5 wt % SiO_2_, unfunctionalized and functionalized with aminosilane, hexamethyldisilazane (HMDS) and triethoxyvinylsilane, respectively. Samples with a thickness of 100–150 µm were prepared and an electric field intensity of 2 kV/mm was applied. As it can be observed from Figure 23, the curves of absorption currents in time for functionalized and untreated SiO_2_ nanocomposites have the same slope, but the currents values are lower for functionalized materials. It was observed that the absorption currents are consistent with the loss tangent behavior in the low frequency region, which strongly suggests that the conductivity could be associated with the interfacial region and/or hydration effects, which are alerted by the enhanced coupling associated with the functionalized materials [102]. Similar results were obtained in other studies [103,104,105,106].

Lau *et al.* [107] investigated the absorption currents behavior of PE nancocomposites unfilled and filled with 2, 5 and 10 wt % nanoSiO_2_, untreated and treated with trimethoxy(propyl)silane coupling agent (C3-treated). The results indicated that the presence of nanoSiO_2_ fillers influenced the values of absorption current. Thus, while the unfilled polymer showed a decrease in time of the absorption currents (in a conventional manner), all the analyzed nanocomposites systems reveal an initial decrease followed by a period in which the current values increase by rising the time of DC field application. It was observed that the time-current characteristics of all analyzed nanocomposites were different from the unfilled polymer and the rate of nanocomposites current values decrease was significantly greater compared to unfilled PE [107]. Using these experimental values, the charge carrier mobility was estimated for unfilled and nanoSiO_2_ filled PE. The results on absorption currents measurements can be used to gain understanding of the relationship between space charge accumulation and movement [107].

#### 6.1.2. Micro/Nanocomposites with Controlled Electrical Conductivity

In many high-voltage and medium-voltage applications such as cables accessories, generator or motor end windings or bushing, many problems with the electrical field stress concentrations can be noticed [108]. In order to avoid breakdown or flashover in these situations, it is necessary to control the electrical field throughout materials with tailored conductivity and non-linear conductivity. These termed field grading materials will reduce the local surface stress in the way which will not exceed the breakdown strength in any location. Even in the past time the field grading materials were used only in AC terminations for medium-voltage applications, nowadays they are involved in both medium and high-voltage applications, under AC and DC conditions because voltage requirements steadily increased and the size of equipment decreased [108].

The main components of a cable are conductor, insulation and grounded shield. In operation, the rated voltage (the potential difference between the conductor and the shield) occurs across the insulation system and the radial stress in this region is non-linear [108]. During the operation process, problems can occur in joints or at the end of the cable due to the fact that the shield has to be removed at cable terminations and the concentrated field in the insulation has to be spread out in a controlled manner in this discontinuity. If this action is not taken into consideration, the electric field stresses may cause flashover problems or breakdown [108]. One solution to solve this problem is called geometrical stress control and it refers to the shield bending and increasing the thickness of the insulation. The major drawback of this solution is the size and cost of the components, particularly in high-voltage applications. Another approach refers to the field grading material, which means that the material possess the ability to distribute the field by itself. Usually, the electrical properties of these materials must be field dependent. In terms of HVAC cables, the field grading materials provide field grading in two ways, either by non-linear resistivity or by capacitive field grading. In latter, the relative permittivity becomes high enough to redistribute the field. For HVDC applications, field grading can be controlled trough a material with field dependent resistivity, which means that the material should become conductive at high fields and stay insulating at low fields [108,109]. In many high-voltage applications, conducting layers are used to obtain equipotential surfaces. Thus, with respect to medium and high-voltage cables, semiconducting layers for levelling and attenuation of high field local values, respectively for reduction of partial discharges phenomena are used. Conducting layers of large radius are commonly tied to the potential of a high-voltage termination to afford some protection from unwanted corona discharges, *etc*. Furthermore, the end windings of high-voltage machines are covered with a semiconducting layer to reduce the partial discharges and leakage [108,110]. As these layers do not have to carry currents, their resistivity does not need to be similar to a metal in order to be effective. For example, a graphite ink is used for rendering cellulose layers conductive as a substitute for metallic foils in the internal stress grading of high-voltage bushing [108]. Similar, semiconducting layers, arranged on both sides of the high-voltage or medium-voltage cable insulation, are manufactured of PE and carbon black or SiC [111,112]. The polymers that are employed for semiconductor layers (acrylates, acetates, PEs, *etc.*) have to exhibit a high thermal stability (up to 250–300 °C during the crosslinking process) to maintain the mechanical properties of the screen and the electrical nature of the inter carbon particle gap [113]. In addition, the polymer materials should comprise a reduced risk for scorching and lumping [112]. Account has to be also taken into the fact that protrusions of the semiconductive layer can push into the insulation and enhance the local electrical field (accelerating electrical aging). The smoothness of a semiconductive material is an important parameter to measure the material consistency and quality. Carbon black has a big influence on the surface smoothness (dispersion of the particles) and on the cleanliness of the semiconductive layers (ions and grit particles) [112]. Semiconductor layers resistivity depends on the polymer matrix nature (recently PE), the concentration of carbon black, process parameters for achieving technological semiconductor layers, temperature, *etc.* [114,115].

In other applications, such as shielding for electronic devices and electrostatic dissipation (ESD), encapsulating, electromagnetic and radio frequency interference (EMI/RFI), thin films coating, packing of electronic circuits, *etc.*, polymeric composite materials with high electrical conductivity are used. The matrixes of these composites are usually based on PE, PVC, PC, PS, epoxy resins, nylon 6.6, acrilonitril-butadien-stiren (ABS), *etc.* As fillers, AlN, carbon and graphite, aluminum, copper, steel or silver particles, polyacrylonitrile (PAN), barium titanate (BaTiO_3_), *etc.*, are applied. [116,117].

Another alternative to impart some conductivity to conventional polymers can be offered by CNTs and nanofibers. As their conductivities can have values in a broad range (from semiconducting to conducting materials), CNTs (single walled carbon nanotubes (SWCNTs) or multi-walled carbon nanotubes (MWCNTs)) can be used in many applications [108]. In literature, several studies on this subject [118,119,120] have been published and one early observation was that the electrical conductivities were not as high as expected given the conductivity of the nanotubes. One example is the study of Cravanzola *et al.* [121] on a piezoresistive sensor device, which has been made by integrating two piezoresistive fibers into two sandwiched PP panels. The fibers were prepared by extrusion from piezoresistive polymeric composites manufactured by melt mixing PP with expanded graphite (EG) and/or MWCNTs (1–2 wt %). It was shown that due to the applied loads mechanical deformation remarkably affected the resistivity of the materials. Haznedar *et al.* [122] investigated composites based on graphite nanoplatelets (GNPs) and/or (MWCNTs)/LDPE and showed the synergistic role of CNTs (1D) and GNPs (2D) in improving the conductive properties of the materials.

Multiple studies regarding the properties of composites demonstrated that percolation phenomenon is very important in controlling electrical properties of these materials, considered as disordered systems [108]. Regarding polymer composites with conducting or semiconducting fillers, there is a critical volume/weight concentration of particles in the matrix, called percolation threshold, above which the electrical or thermal conductivity increases suddenly, as a result of a continuous conductive path formation [108].

In classical percolation, theory applied to composites a physical connection between the filler particles and the conductivity (σ) near the percolation threshold can be described by the power law:

σ ∝ (φ − φ_c_)*^t^* (3)

where φ is the volume fraction of filler, φ_c_ is the percolation threshold and *t* is a power law constant that depends on the geometry of the system [108,116,122]. φ_c_ is also a function of the filler geometry, dispersion and the type of connectivity between particles (*i.e.*, tunneling *versus* Schottky barrier) [62]. Typical values for three dimensional systems are *t* = 2.0 and φ_c_ = 0.17 for spherical particles [108].

The model requires some modifications due to the filler geometry, dispersion and conduction in nanoparticles filled polymers. In the case of polymers nanocomposites, there can be a very thin polymer layer completely encasing the nanofillers that prevents direct particle-particle contact. In this case, electrical percolation occurs when particles are close enough for tunneling conduction through the interstitial layer (*i.e.*, carbon black filler polymers) [108]. In the case of spherical nanofillers that are not perfectly dispersed in the polymer matrix, there are resulting fractally and not necessarily spherical agglomerates during synthesis and processing. This situation will shift the percolation threshold because the aspect ratio of nanofillers is greater than one or they effectively fill a larger volume because of the fractal shape. Thus, φ*_c_* could be predicted by using the concept of an excluded volume, which is the volume around the filler that cannot be occupied by the center of another object [108]. Due to the fact that CNTs tend to bundle, their shape remaining cylindrical, a hard core model is the most appropriate to estimate the percolation threshold.

Thus, nano/microfillers offer the ability to tailor and optimize the electrical properties of polymers, but their commercial use meets challenges such as a poor dispersion performance during large scale processing and understanding the electrical and thermal conduction mechanisms.

#### 6.1.3. Relative Permittivity and Loss Factor

Many research studies were performed on the relative permittivity and loss factor of micro/nanocomposites materials [123]. If several tens weight percentage of inorganic microfillers are introduced in polymers, usually the relative permittivity of the composite increases [33]. This is because fillers have a higher permittivity by nature compared to the base polymers and they cause Maxwell-Wagner interfacial polarization, which provide information about charge trapping associated with internal surfaces and relaxation processes associated with dipole reorientation [124]. This type of polarization will increase the values of loss tangent, too [33]. Increased values for microcomposites are usually explained in the terms of the Lichtenecker-Rother logarithmic law of mixing [33]. Conversely, the addition of nanoparticles causes major changes in the dielectric response and their permittivities were found to decrease in many cases, such as Figure 24a,b from Nelson *et al.* [22] experimental studies.

At high frequencies, the microcomposites showed a higher relative permittivity, probably, due to the higher permittivity of fillers incorporated in the base polymer (ε_r_(TiO_2_) ≈ 99) [22]. For example at 1 kHz and 393 K, the measured relative permittivities were 9.99 for the base resin, 13.8 for microcomposites and 8.49 for nanocomposites, which is significantly less than the base polymer. This result suggests that the interaction zone, which surrounds the nanoparticles, has a profound effect on the dielectric behavior of nanocomposite and gives rise to limited cooperative movements of dipolar reorientation within them [22,124]. This behavior could be also due to the movement restriction of epoxy molecules end-chains of side-chains by the presence of nanoparticles [22].

In the mid-range of frequency between 0.1 and 100 Hz, the base resin and the nanocomposites show the same behavior with a small dielectric relaxation, probably due to the bound water [22,124]. The real permittivity of the microcomposite shows a significant increase with decreasing frequency associated with Maxwell-Wagner polarization [22].

At low frequencies, the nanocomposite materials show a different behavior. The slope of the real part changes from −2 to −1 in these Bode plots and the loss tangent is flat and independent of frequency and this can be explained by the “low-frequency dispersion” (LFD) proposed by Jonscher [125] or what Dissado and Hill [126] refer to as “quasi-DC” (QDC) behavior. This behavior is observed when charge carriers have some limited freedom of movement within the material and they may follow tortuous paths under the influence of the electric field, that do not allow complete transport through the material [22]. Since nanoparticles could cause morphological changes to the epoxy resin during the crosslinking process, a “dielectric interaction” layer surrounding these particles could be formed. Lewis [79] has considered the electrical (polarization and conduction) phenomena in the zones that are surrounding the nanoparticles and the formation of a charged layer (Stern layer) on the surface of the particles, encircled by a diffuse charged layer (Gouy-Champan layer) [76,127]. The Gouy-Champan layer is highly conductive compared to the polymer base and charge movement through it would be relative facile [22]. If these layers overlap between several nearby nanoparticles, charge movement over limited distances will be facilitated and the path-lengths of such carriers would form a distribution [22]. Dissado and Hill [126] modeled this field-enhanced percolation in the terms of fractal circuits. Therefore, the reduction of the fillers concentration from 10 to 1 wt % did not bring fundamental changes, but nanocomposite materials showed a low frequency response, more typical of the base polymer and microcomposites, suggesting that behavior changing requires filler concentrations higher than a few percent [22].

Singha *et al.* [128] analyzed the dielectric behavior of epoxy nanocomposites with single nanofillers of Al_2_O_3_ and TiO_2_ at low filler concentrations (0.1/0.5/1/5 wt %) over a frequency range of 1 MHz–1 GHz. The experimental results obtained on these nanocomposites samples showed very different dielectric characteristics compared to those for microcomposites. In the case of polymer microcomposites the permittivity was increasing by rising the filler concentration, but for a certain concentration of nanofillers into the polymer and depending on their permittivity value, the equivalent permittivity of the epoxy nanocomposite is smaller compared to the one of the base resin, for all the measured frequencies. These results suggest that there is a strong dependence of the filler permittivity and concentration on the equivalent permittivity of the nanocomposite material for all the analyzed frequencies. The loss tangent behavior was not affected by the filler concentrations, but in the case of epoxy/Al_2_O_3_ nanocomposites, loss tangent values were found to be marginally lower at all concentrations when compared to the values for unfilled polymer [128].

Kochetov *et al.* [129] realize a study on the dielectric spectroscopy of epoxy-based nanocomposites filled with different types of particles, such as Al_2_O_3_, AlN, MgO, SiO_2_ and BN. The nanoparticles surfaces were modified with a silane-coupling agent, in order to realize the compatibility between the inorganic and organic components and to obtain a better dispersion of the nanofillers into the polymer matrix. The relative permittivity of nanocomposites shows an unusual behavior. It was observed that the introduction of a low percentage (below 5 wt %) of high permittivity filler results in a decrease of the bulk polymer permittivity. This can be explained by the presence of the interface layer of surface modified particles, which plays a more important role than the nature of the particles and also by the immobilization caused by the surface treatment of the nanoparticles [129]. It was observed that dielectric losses in the system do not change significantly with the addition of nanofillers up to 5 wt. % [129]. Similar studies on dielectric behavior of micro/nanocomposites systems based on epoxy resin and different types and concentrations of filler were performed by Mackersie *et al.* [123], Fothergill *et al.* [124,130], Tanaka *et al.* [4,33], Singha *et al.* [32,128], Smith *et al.* [104], Plesa *et al.* [131], Kozako *et al.* [132], Castellon *et al.* [26], Heid *et al.* [133], Mo *et al.* [134], *etc.*

**Figure 25:**
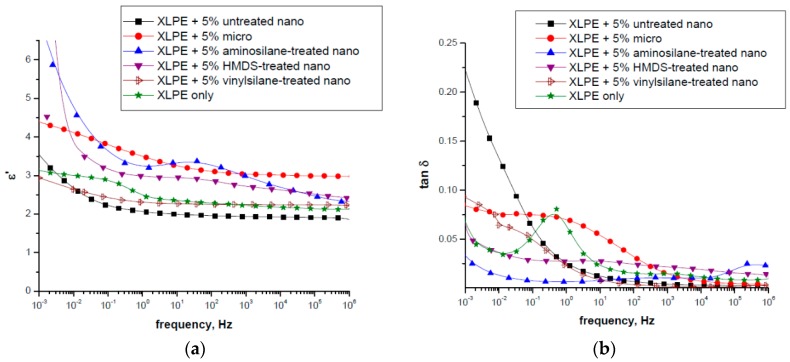
(**a**) Real part of relative permittivity and (**b**) loss tangent of functionalized XLPE at 23 °C (© 2016 IEEE. Reprinted, with permission, from [102]).

Roy *et al.* [102,135] analyzed the dielectric behavior of different systems based on XLPE/SiO_2_ functionalized with amino-silane, hexamethyl-disilazane (HMDS) and triethoxyvinylsilane agents. The dielectric spectroscopy analyses (see Figure 25a,b) provide considerable insight into the nature of the structure, which contributes to the polarization and loss. The results showed that the untreated nanocomposites exhibit a relative permittivity lower than the unfilled polymer, which suggests the presence of an interfacial zone around the particles with a smaller permittivity compared to the bulk polymer [102]. A marked dispersion was observed in the case of unfilled XLPE at the frequency of 1 Hz, but was eliminated for the cases of functionalized nanocomposites. With respect to the loss tangent, it is very significant that a QDC conduction region appears in the case of untreated nanoparticles, which suggests the presence of a conductive interface in their case [102]. Low frequency dispersion can be observed in microcomposites which is absent in all nanocomposites. This behavior likely results from Maxwell-Wagner polarization, which is mitigated for the nanodielectrics [102]. Similar studies on dielectric behavior of micro/nanocomposites systems based on polyethylene and different types and concentrations of filler were performed by Ciuprina *et al.* [83,136,137,138], Tanaka *et al.* [24], Panaitescu *et al.* [139], Plesa [84], Hui *et al.* [140], Lau *et al.* [53], *etc.*

In other systems such as PI/SiO_2_ materials [141], loss tangent tends to decrease for pure PI, PI/SiO_2_ microcomposites and PI/SiO_2_ nanocomposites, at low frequency region, up to 200 Hz. Regarding PI/SiO_2_ microcomposites, a peak appears in the middle frequency region (about 1 kHz) due to the Maxwell-Wagner interfacial polarization and is more much reduced in terms of PI/SiO_2_ nanocomposites. This peak can be caused by the mitigation of the field around fillers due to their size differences [4,141]. Room temperature vulcanized (RTV) silicone rubber/LS nanocomposites exhibit a slight increase of loss tangent and a slight decrease of relative permittivity at the industrial frequency [4]. Evaluation of the dielectric behavior seems to be more complicated when comparing neat polymer, microcomposites and nanocomposites materials [4]. The most important aspect to be clarified is whether or not relative permittivity and loss tangent are reduced by nanomization at the industrial frequencies. In literature, some reported data indicate a certain reduction whilst other data did not, which creates confusions [4]. These results can depend on many factors, such as how inorganic and organic components are compatibilized, the dispersion of the fillers in the base polymer, fillers agglomerations, humidity, temperature, *etc.*

#### 6.1.4. Partial Discharges and Erosion Resistance

The resistance of insulating materials to partial discharges (PD) is a very important property for high-voltage applications, such as the stator end windings of rotating machines or wires of randomly wound motors or HVDC XLPE cables, where PD will gradually erode the insulating materials and cause breakdown [25]. PD resistance of polymer insulation can be evaluated by using several configurations of electrode systems such as International Electrotechnical Commission (IEC) electrode and rod-to-plane electrode systems. The former gives surface roughness, while the latter allows evaluation of erosion depth and can be used for micro/nanocomposites materials characterization [142].

**Figure 26:**
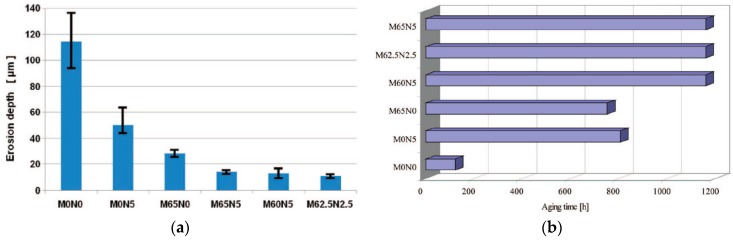
(**a**) Partial discharge erosion depths measured on neat epoxy resin (M0N0), 5 wt % nanoSiO_2_ (M0N5), 65 wt % microSiO_2_ (M65N0), 65 wt % microSiO_2_ + 5 wt % nanoSiO_2_ (M65N5), 60 wt % microSiO_2_ + 5 wt % nanoSiO_2_ (M60N5), 62.5 wt % microSiO_2_ + 2.5 wt % nanoSiO_2_ (M62.5N2.5) epoxy composites aged at 4 kV/600 Hz for 60 h; (**b**) time to failure of epoxy composites materials with 10 kV/250 Hz applied (© 2016 IEEE. Reprinted, with permission, from [25]).

Krivda *et al.* [25] evaluated the resistance to erosion due to PD of the epoxy micro/nanocomposites mixtures, using a rod-to-plane electrode system (see Figure 26a,b). From the results showed in Figure 26a, it became clear that a combination of micro and nano-sized fillers in epoxy composites provided better protection against PD erosion than the base resin, composites containing either solely microfillers or solely nanofillers [25]. Due to the fact that results depend on the test conditions (Figure 26a) shows the results obtained at 4 kV/600 Hz and Figure 26b shows the results at 10 kV/250 Hz) it is impossible to identify the best combination of micro and nanofillers in the polymer matrix. Nevertheless, from the obtained results is clear that micro+nanocomposites had smaller erosion depths and longer times to failure compared to unfilled resin and micro/nanocomposite materials [25]. When the material is only filled with microparticles, there is a relatively large volume of neat epoxy that is exposed to PD and degrades much faster compared to inorganic fillers. Inorganic particles can easily withstand temperatures above 1000 K, whereas epoxy thermally decomposes at 600 K and gives rise to large erosion depths in microcomposite materials. When nanoparticles are added into the mixture, they are filling the space between microparticles, creating additional barriers to PD. In addition, when the epoxy resin layer from the top is degraded, nanoparticles are released, but remaining on the surface, provide an additional PD protection of the composite [25].

Many experiments were done to investigate PD resistance of epoxy micro/nanocomposite materials. Most of them demonstrated that the addition of nanoparticles could improve this electrical property, despite of using the epoxy without fillers [143].

Iizuka *et al.* [144] analyzed two types of epoxy/ SiO_2_ nanocomposites, such as Aerosil (prepared by dispersing commercially available nanoSiO_2_, termed Aerosil, in epoxy resin and by curing the whole mixture) and Nanopox (prepared by directly curing available mixture of epoxy and nanoSiO_2_, termed Nanopox) in order to clarify the effect of nanofiller dispersion and coupling agents on the electric filed endurance. It was found that partial discharges resistance was improved only by adding nanofillers and coupling agents [144].

Tanaka *et al.* [145] investigated PD endurance of epoxy and 1, 2, 3, 4, and 5 wt % SiC nanocomposites in comparison with that of epoxy/SiO_2_ nanocomposites. It was remarked that epoxy resins could be improved in their PD erosion performance by replacing SiO_2_ with SiC nano fillers, while the erosion profile was narrow in epoxy/SiO_2_ nanocomposite. It can be concluded that SiO_2_ fillers remain more stuck on the surface after exposure to PD than SiC fillers [145].

Preetha *et al.* [146] analyzed PD characteristics of epoxy nanocomposites samples with a good dispersion of Al_2_O_3_ particles (0.1, 1, 5, 10 and 15 wt %) into the polymer matrix. PD experiments were conducted at 10 kV for different durations using IEC type electrodes. The results were compared to unfilled epoxy and epoxy microcomposites. It was observed that even for a concentration of 0.1 wt % Al_2_O_3_ nanoparticles, the PD resistance of nanocomposite improved considerably. It was observed that the inter-particle distance has a significant effect on the discharge resistance to degradation and the improvements are attributed to the interactions between nanoparticles and the epoxy chains [146].

**Figure 27:**
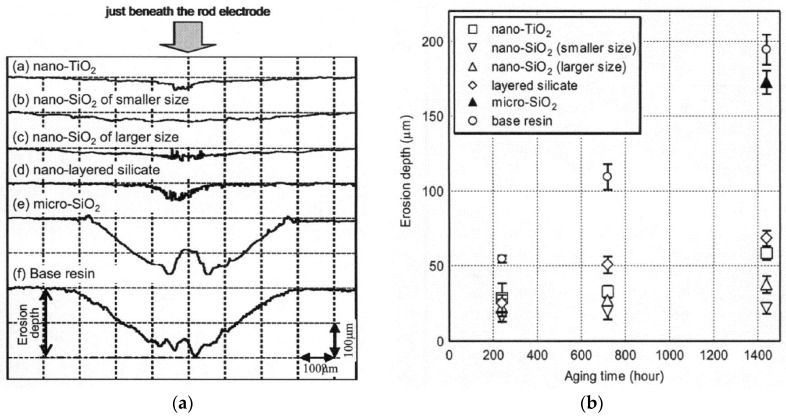
(**a**) Surface profiles of erodated areas due to PDs in the samples containing different types of micro/nanofillers and without fillers after 120 h adding at 720 Hz; (**b**) Temporal change in erosion depth of area eroded by PDs at 4 kV of 720 Hz (© 2016 IEEE. Reprinted, with permission, from [148]).

Kozako *et al.* [147,148] investigated PD for four types of epoxy nanocomposites with nanoTiO_2_, two different sizes of SiO_2_ and LS, unfilled epoxy and filled with microSiO_2_ (see Figure 27a,b).

Li *et al.* [149,150] analyzed PD erosion resistance of different kinds of insulation samples, such as neat epoxy, epoxy/5 wt % nanoAl_2_O_3_ composite, epoxy/60 wt % microAl_2_O_3_ composite, and combined epoxy/2 wt % nano- with 60 wt % micro-Al_2_O_3_ composite, using a rod-to-plane electrode system. It was observed that nanocomposites take the longest breakdown time (307 min) compared to neat epoxy (186 min), microcomposite (94 min), and micro/nanocomposite (275 min) [143]. From all these experimental results, it was concluded that by adding a low concentration of nanofillers into epoxy resin matrix, PD are significantly improved [143]. This is most likely due to the strong bonding between nanoparticles and epoxy resin chains at the interface zone, which causes a speed reduction of the material local degradation [32]. Addition of microfillers does not make any significant contribution to PD resistance compared to nanofillers, but they can increase the thermal conductivity, which is an advantage [149]. Similar results were obtained by Henk *et al.* [20], Li *et al.* [151], and Zhang *et al.* [152], Imai *et al.* [153].

**Figure 28:**
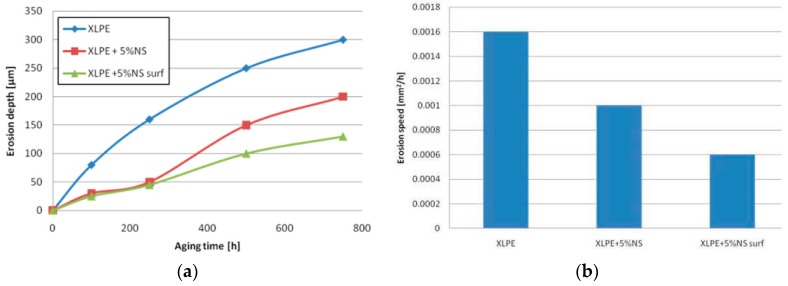
(**a**) Evolution of PD erosion depth with aging time of unfilled XLPE, XLPE with 5 wt % unfunctionalized nanoSiO_2_ and XLPE with 5 wt % chemical agent functionalized nanoSiO_2_ and (**b**) erosion speed for these kinds of XLPE (© 2016 IEEE. Reprinted, with permission, from [24]).

The available results and data for XLPE polymer with nanofiller are limited in literature. Tanaka *et al.* [24] reported evidence of the enhanced of XLPE nanocomposite PD resistance values (see Figure 28a,b). The analyzed samples were based on standard commercial XLPE, in order to have more impact on improving the current insulation used for power-extruded cables.

Two methods of PD resistance evaluation were conducted in this investigation: the first by using a rod-to-plane electrode and the second similar to the IEC electrodes system. The first method showed that partial discharges endurance was significantly improved in the case of XLPE with 5 wt % SiO_2_ nanofillers (chemically surface functionalized) compared to unfilled XLPE (see Figure 28a,b) [24].

**Figure 29:**
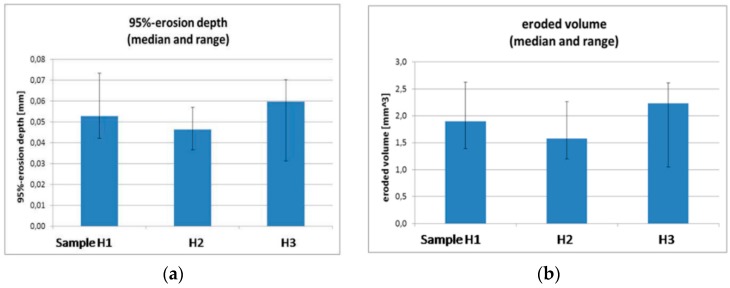
(**a**) PD erosion depth and (**b**) PD erosion volume for unfilled XLPE (sample H1), XLPE/5 wt % unfunctionalized nanoSiO_2_ (sample H2) and XLPE/5 wt % functionalized nanoSiO_2_ (sample H3) (© 2016 IEEE. Reprinted, with permission, from [24]).

On the other hand, with the second method, which uses an electrode similar to the IEC electrode system to test the three heat-treated samples (unfilled, filled nanoSiO_2_ without and with surface-treated filler), no apparent improvement was observed by the addition of nanofillers (see Figure 29a,b). It was generally speculated that this is due to the effect of the filler treatment of the samples, but the test method and data analysis should be further investigated [24,143].

Experimental studies were also performed on other type of systems. Ansorge *et al.* [154] analyzed the influence of various fillers (alumina trihydrate (ATH), Al_2_O_3_ and SiO_2_ with different sizes (from 0.3 to 18 μm)) and their surface modifications (by the material supplier and *in situ* during compounding) on the erosion resistance of high temperature vulcanized (HTV) silicone rubber (SR) composites. It was found that with respect to ATH particles, larger particles showed slightly better results than smaller ones, due to the formation of boehmite [AlO(OH)], which causes a release of the bound water if the temperature exceeds certain values. The particles modifications with vinyltrimethoxysilane (VTMS) and methyltrimethoxsilane (MTMS) improved not significantly the erosion resistance of the composites, but reduced the water-uptake. It was concluded that to achieve a low erosion rate, high- filler loadings are necessary [154]. Heid *et al.* [133] found that incorporation of hexagonal boron nitride (h-BN) particles in epoxy resin resulted in significant improvements of parameters such as resistance to PD. Other studies showed that PD resistance improves in PI by nanostructuration with LS [33] and PD resistance was larger in PI/SiO_2_ nanocomposites than pure PI [4]. In summary, it is indicated that nanomization improves PD resistance of polymers, but depends also how nanofillers are dispersed in the polymer matrix and the compatibility between organic and inorganic components [4].

#### 6.1.5. Space Charge Accumulation

**Figure 30:**
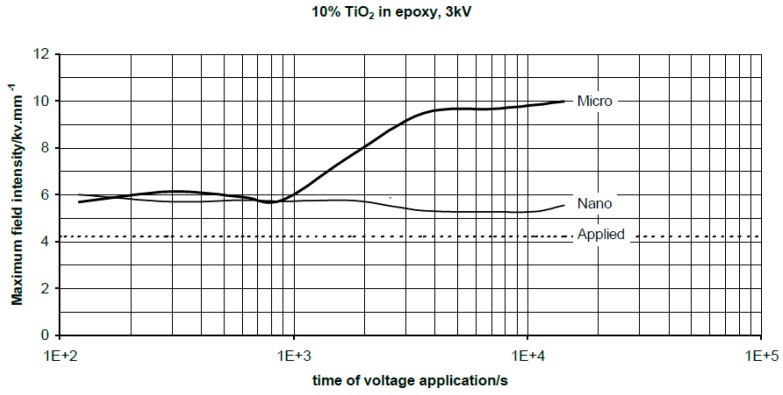
Maximum field intensity as a function of voltage time application on epoxy/TiO_2_ micro/nanocomposites (© IOP Publishing. Reproduced with permission. All rights reserved [22]).

Space charge occurs in a dielectric material when the rate of charge accumulation is different from the rate of removal and arises due to the moving or trapped internal charges, such as electrons, holes and ions [155]. It is generally undesirable since it causes a distortion in the electric field, increasing the internal field locally within the insulator, which will lead to a faster and premature failure of the material [124,155]. Thus, the homocharge (charge near an electrode of the same polarity as the electrode originally in contact with it) decreases the electric field in the electrode vicinity. As a result, a concomitant increase of the electric field elsewhere in the insulator volume is undesirable. This increase can lead to an intensification of partial discharges in the insulating system which results in an acceleration of the material electrical degradation process and a reduction on its lifetime [124]. Heterocharge increases the electric field next to the electrode, so a reduction of space charge accumulation is therefore an important goal [124]. Thus, the mechanisms of space charge formation are considered as a determination factor in establishing the overall dielectric properties of a polymeric insulation system and they are very complex in comparison to many other types of materials [155]. In semicrystalline PE, the interfaces between the crystalline and amorphous phases are associated with the presence of charge trapping sites, which are likely to influence the charge accumulation. By the addition of nanofillers, the charge transport mechanism will become much more complicated because fillers will introduce numerous interfaces and interactions between the polymer and nanofillers. The presence of such interfaces will introduce/modify the distribution of the trapping sites within the system and the charge transport mechanisms will be affected [155]. Space charge is usually measured by different methods, such as piezoelectric induced pressure wave propagation (PIPWP) method, laser induced pressure propagation (LIPP) method, thermal step method (TSM) and pulsed electro-acoustic (PEA) method. Being aware of space charge density by different analytical and numerical methods, the electric field distribution and its maximum field values can be calculated [33]. The early experimental work regarding the space charge accumulation in nanocomposites was reported in comparison with microcomposites [22]. Nelson *et al.* were the first in reporting the reduction of space charge density through nanocomposites compared to microcomposites based on epoxy resin and TiO_2_ [22]. Figure 30 demonstrate the difference between nanocomposites and microcomposites based on epoxy resin with 10 wt % of TiO_2_, with the average diameter of 23 nm/1.5 µm and shows the maximum field intensity (at that point in the sample, where the electric field is highest) as a function of time for the two systems [22].

Accordingly to these analyses, it was observed that the maximum field in microcomposites builds up to over twice the average applied electric field, whereas nanocomposites stabilize at the field just a little bit higher than the average [33]. Trials were made in order to assign the polarity of charge formed in composites, but they were not conclusive if the charge formed near the electrodes is homo or hetero, due to the complicated distribution and conditions that affect space change [33].

In literature, it was reported that space charge is mitigated by nanostructuration in different nanocomposites systems, such as epoxy/TiO_2_, Al_2_O_3_ and ZnO, PP/EVA layer silicate and LDPE/TiO_2_ [33]. Yin *et al.* [156] analyzed nanocomposites of LDPE with TiO_2_, prepared via solution blending method. The space charge distribution of the samples with and without nanoTiO_2_ was measured with PEA method. It was found that hetero-polar space charge near electrodes was much less in LDPE/TiO_2_ nanocomposites compared with pure LDPE under lower DC stress, no more than 40 kV/mm [4,156]. The space charge inside the nanocomposites was much more uniform compared to the base polymer, which means that electrical stress concentration was improved under DC stress in nanocomposites [4,156]. Another observation was that the decay rate of the space charge remnant in LDPE/nanoTiO_2_ increased by increasing TiO_2_ concentration, when short-circuited after pre-stress at 50 kV/mm for 1 hour [4,156].

**Figure 31:**
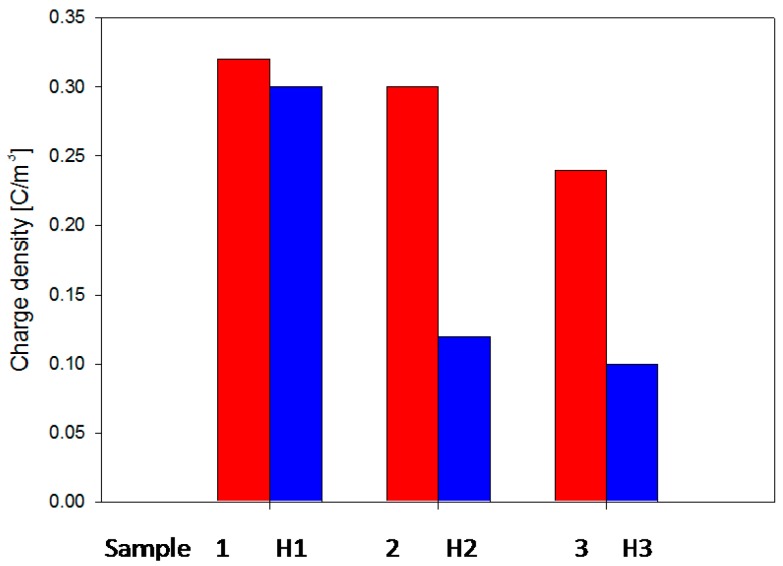
Space charge distribution at 20 kV/mm in unfilled and filled XLPE (samples 1, 2 and 3) before and after treatment (samples H1, H2 and H3) at 80 °C for five days (© 2016 IEEE. Reprinted, with permission, from [24]).

CIGRE Working Group D1.24 [24] realized a comprehensive experimental investigation of XLPE and its nanocomposites with fumed SiO_2_. The research studies were carried out in different countries, but all the samples were prepared by only one source and evaluated by experts from several laboratories [24]. Three types of samples were analyzed involving unfilled XPLE (standard commercial material used for extruded power cables—sample 1), XLPE with 5 wt % unfunctionalized nanoSiO_2_ (sample 2) and XLPE with 5 wt % functionalized nanoSiO_2_ (sample 3). The samples were vacuum heat treated at different temperature values and time durations. Two types of measurements systems were applied to measure space charge in XLPE samples, such as PEA method and thermal step (TS) method at high/low field, by different research teams [24]. One of the results revealed that the lowest space charge amount is obtained when the nanofiller is surface treated (see Figure 31).

The overall results showed that heterocharge is generated for unfilled XLPE and is due to some cross-linking residues and natural impurities [24]. On the other hand, it was confirmed that nanofillers reduce this heterocharge since nanoparticles are characteristic for impurities absorbance. Concerning the charge injection, it was shown that homocharges are easier injected into filled than unfilled XLPE and when nanofillers are added, a charge compensation takes place. It was also highlighted that charge packets appeared for very high electric field values (near breakdown) [24]. This unstable and chaotic phenomenon consists of a slowly travel across the insulator of some waves of charges, with a rate of about 1 mm/hour and a magnitude that can double the local electric field. These charge packets are generally found to be reduced by the addition of nanoparticles [124]. Similar findings were obtained also by Lau *et al.* [155] on nanocomposites based on XLPE with 2 wt %/5 wt %/10 wt % treated and untreated nanoSiO_2_.

In other polymer systems, such as EVA and i-PP, charge accumulations were considerably large at the field value of 60 kV/mm, but tended to decrease by increasing the addition of nanofillers from 2 to 6 wt % [33]. A common behavior is the charge reducing at high electric field due to nanofillers introduction, while it is increasing at low field due to ionic impurities included in nanofillers. This charge can be swept away by the pretreatment of nanofiller purification [33].

In summary, the following findings are confirmed as effects of nanostructuration: space charge increases at low fields and decreases at high field; space charge inception field decreases; space charge is generated internally and charge decay time decreases [124].

#### 6.1.6. Electrical Breakdown

**Figure 32:**
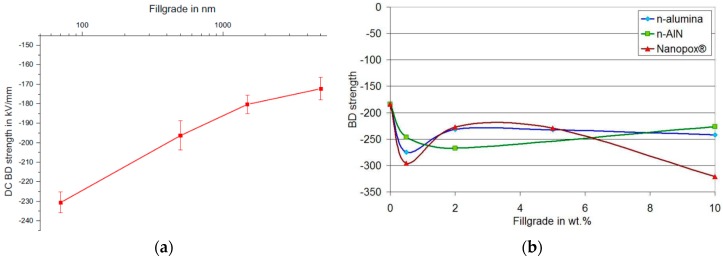
(**a**) Short term DC breakdown strength for BN/epoxy resin composites as a function of filler size (© 2016 IEEE. Reprinted, with permission, from [30]) and (**b**) Weibull scale parameter, which shows the voltage for 63.2% failure probability of samples with two components (epoxy resin and nanofillers) (© 2016 IEEE. Reprinted, with permission, from [157]).

Electrical breakdown of insulating materials is an important factor in high-voltage applications [59]. The incorporation of inorganic fillers into the base polymer can significantly modify the electrical breakdown of the composite material, depending on the filler concentration, their shape, size and surface modifications with different agents, materials homogeneity affected by the dispersion of the fillers into the base polymer, and the electrical properties of the fillers [59]. In order to obtain high electrical breakdown strength of composites is it necessary to choose fillers with similar electrical characteristic as the polymer matrix, since electric field distortion and enhancement can be caused by the differences in relative permittivity and electrical conductivity between inorganic fillers and organic polymers [59]. The dielectric strength of the polymers can be deteriorated by high-permittivity fillers (BaTiO_3_, SiC, ZnO and AlN) and high electrical conductivity fillers (carbon black, carbon fiber and nanotubes, graphite, metals), but they can be used in applications demanding high thermal conductivity materials [59]. Low permittivity and high electrical resistivity fillers can be used in polymer composites with high thermal conductivity and high breakdown strength for the insulation systems of electrical equipment. For example, combining BN nanofiller (70 nm, 10 wt %) with microfiller (500 nm, 1.5 μm, 5 μm, 10 wt %), Andritsch *et al.* [30] demonstrated an increase in the DC breakdown strength of an epoxy composite. From Figure 32a it can be observed that the increase coincides with the increase of the interfaces in the composite material [30]. It is suspected that one of the key parameters in improving the dielectric behavior of nanodielectrics refers to the strong interfaces between polymers and fillers, because of their surface modifications [30]. Even when the functionalization of nanoparticles was not performed, surprising results were obtained [30].

The electrical breakdown strength values were measured in the case of epoxy-nanocomposites with Al_2_O_3_, SiO_2_ and AlN as-received/functionalized and plots for a Weibull scale parameter can be seen in Figure 32b, which shows the voltage for 63.2% failure probability of the samples [157].

Electrical breakdown results are frequently analyzed through Weibull statistics (Weibull plots) and represent the cumulative probability of breakdown, which would equate to the proportion of specimens failed for a large sample size [124]. Since these composites had their highest DC breakdown strength for 0.5 and 2 wt % fillers concentrations, it can be assumed that also epoxy resin/BN composites with surface functionalized particles of 70 nm on average would also have higher breakdown strength for fillers concentration between 0.5 and 2 wt % [30]. Other studies [4] reported that electrical breakdown values remained almost the same up to 10 wt % nanoparticles concentration, while it decreased significantly for 10 wt % microparticles loading.

**Figure 33:**
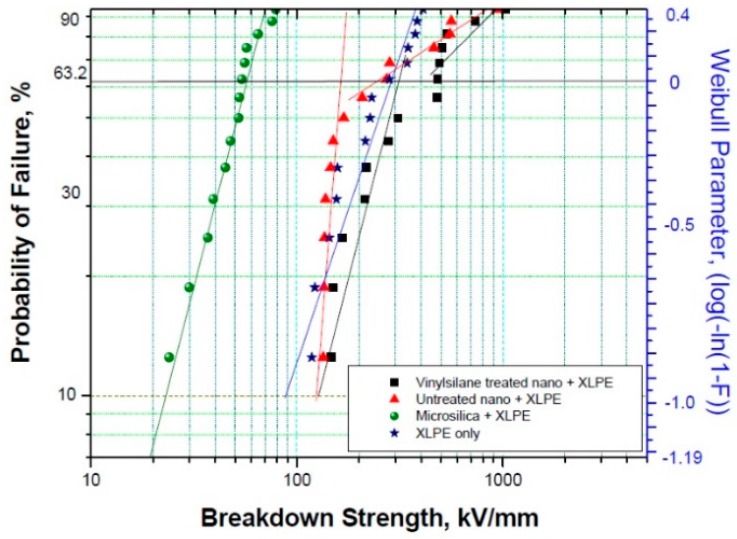
Weibull plot for the electrical breakdown strength of XLPE with 5 wt % micro-/untreated and vinylsilane-treated nanoSiO_2_ at 25 °C (© 2016 IEEE. Reprinted, with permission, from [135]).

Roy *et al.* [135] observed that incorporation of SiO_2_ nanoparticles into XLPE increased the dielectric strength significantly compared to the incorporation of microparticles and their values were compared with the base polymer in Figure 33.

A dramatic increase in breakdown strength was observed in the case of untreated nanocomposites compared to microcomposites. However, the largest increase was observed for the vinylsilane treated SiO_2_/XLPE composites at 25 °C that maintained to increase their values at elevated temperature of 80 °C. For all the analyzed samples, the Weibull shape parameter (β) increased at 80 °C due to an increase in free volume with temperature [135]. Electrical breakdown of polymer-based micro/nanocomposites is affected by several factors, such as degree of crystallinity, space charge accumulation, interfacial area, temperature, free volume and type of bonding [135]. In this study [135], the highest increase in electrical breakdown was achieved with nanoparticles compared to microparticles, where no significant change in crystallinity occurred. It has been postulated that nanoparticles prevent the space charge accumulation in the volume of nanocomposites, by generating local conducting paths. The existence of these paths through the overlapping of nanometric double layers, can explain the breakdown values [135]. Similar results were obtained by Lau *et al.* [155] in their experimental studies.

**Figure 34:**
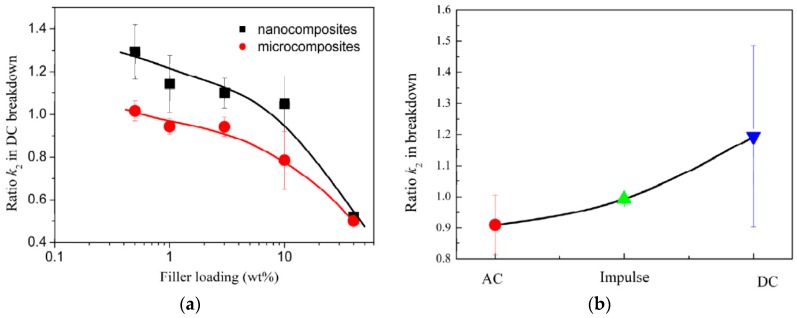
Ratio *k*_2_ (**a**) in DC electrical breakdown *versus* micro/nanofillers concentration and (**b**) of nanocomposites in electrical breakdown depending on the AC and DC applied voltage (© 2016 IEEE. Reprinted, with permission, from [151]).

Li *et al.* observed in their review [151] that DC breakdown strength decreases for micro/nanocomposites by increasing the filler loadings as illustrated in Figure 34a. In order to effectively comment on these published experimental data in the review [151], the ratio *k*_2_, between the breakdown voltage values of nanocomposites with different fillers concentration and the ones of the polymer matrix are employed. Below a certain content (about 10 wt %) nanofillers indicate a positive effect on improving DC electrical breakdown strength [151]. From Figure 34a it can be observed that microfillers have a negative effect on DC electrical breakdown [151].

Calebrese *et al.* [158] demonstrated that nanoparticles exhibit both increasing and decreasing of breakdown strength in different systems. In obtaining these variations, the effect of processing can also be a determining factor. For example, it was shown that both microfillers and size agglomeration of nanoparticles, lead to reductions in the breakdown strength [158]. Tanaka *et al.* [4] observed that DC breakdown strength was enhanced for nanocomposites based on PP, while it did not changed significantly in the case of EVA copolymers.

Electrical breakdown property of dielectrics depends also on the applied voltage [151]. It was observed that in the process of data compilation, electrical breakdown field stress presents a strong dependence on the applied voltage as depicted in Figure 34b [151]. This figure clearly reveals that nanofillers are beneficial to improve electrical breakdown strength of unidirectional voltage, which was affected by space charge [151].

In other nanocomposites systems based on epoxy resin/ZnO and epoxy resin/layers silicate, better performance in AC breakdown strength were reported [33]. It was shown that the addition of a very small amount of ZnO nanoparticles (between 0.5 and 1 wt %) in epoxy resin, leads to a significant improvement in breakdown time [33].

Therefore, electrical breakdown strength might not be significantly affected by nanomization, under small concentrations of nanofillers and proper dispersion conditions. Favorable results were reported in the cases presented before in comparison to unfilled and micro-filled materials [4].

#### 6.1.7. Tracking Resistance

In high-voltage applications, especially on outdoor polymeric insulators appear tracking phenomena, which means the formation of permanent conducting path across the insulator surface due to surface erosion under voltage stress. During the service, the outdoor polymeric insulators are coated with dust, moisture or environmentally impurities, leading to conducting path formation on their surface. When a voltage is applied, this path will start to conduct, resulting in heat generation and sparks occurrence, which damage the surface of the insulator. Due to the fact that polymers are organic materials, the carbonizes regions at the sparking places act as semiconducting or conducting channels resulting in an increased stress in these regions and over the rest of the material. Consequently, the temperature increases in the vicinity of the channels and a new region is carbonized. As the process is cumulative, the channels increase in time and the carbonized track bridges the entire distance resulting in a failure of the insulation [159].

One common approach for increasing the tracking resistance of polymeric insulators, corresponding to an increase of equipment lifetime, is the introduction of inorganic microfillers into the polymer matrix. In this direction, Piah *et al.* [160] analyzed the tracking resistance through experimental observations of leakage currents values and carbon track development of linear low density polyethylene and natural rubber (LLDPE/NR) blends with and without ATH fillers. The experimental results showed that the compound of 80% LLDPE mixed with 20% NR, without ATH seems to be the best compound based on the least damage and the lowest normalized degradation index [160]. Although the experimental results revealed favorable tracking resistance enhancement, for high-voltage applications, this property of polymeric insulators should be further improved to attain greater reliability [159] and nanocomposites materials could be the answer.

**Figure 35:**
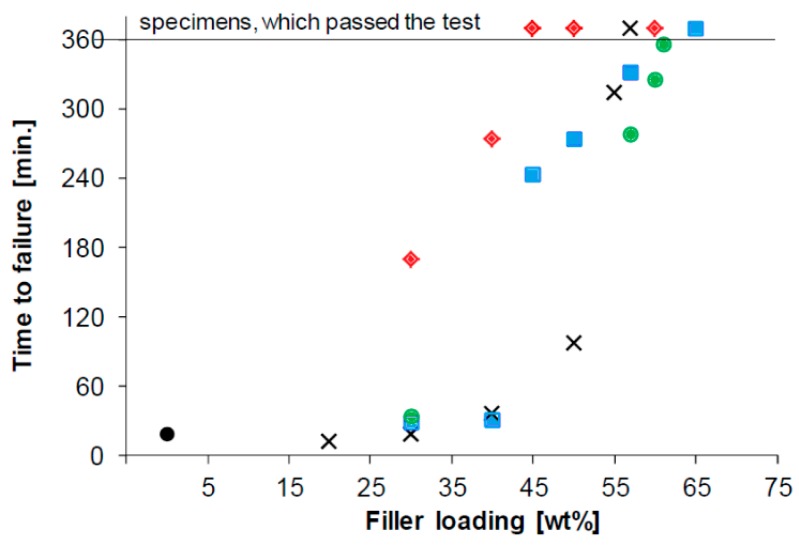
Average time to failure of composites material supplier (crosses), ground modified ATH of 3.5 µm (squares), ground modified ATH of 3.5 µm (diamonds) and surface modified ground SiO_2_ particles (circles) in the inclined plane test (IPT) at 6 kV filled at different contents and compared with unfilled base rubber (black circle) (© 2016 IEEE. Reprinted, with permission, from [154]).

Tanaka [33] considers that silicone elastomers, which can be used for outdoor insulators are expected to have better tracking resistance performances by nanostructuration, but further investigations need to be accomplished in this direction. By addition of ATH nanoparticles in RTV silicone rubber, the tracking performances of the silicone rubber are improved [4]. El-Hag *et al.* [161] obtained experimental results on the erosion resistance of silicone rubber (SIR) filled with 12 nm size fumed nanoSiO_2_ and filled with 5 µm size SiO_2_ microfiller. It was concluded that the erosion resistance of SIR materials increased by increasing the percentage of fillers, and similar performances were observed for 10 wt % nano-filled SIR and 50 wt % micro-filled SIR. Sarathi *el al.* [162] demonstrated that the tracking time is higher in terms of aged epoxy nanocomposites compared to pure epoxy. Ageing studies were carried out to understand the surface characteristic variation through contact angle measurements. Raetzke *et al.* [163] tested the resistance to tracking and erosion of silicone rubber with two different kinds of nanoscale SiO_2_ filler particles. The results showed a high improvement of the resistance to both stresses for one type of untreated SiO_2_ particles at very low filler contents. Later, in 2015, Ansorge *et al.* [154] analyzed the influence of various fillers, such as ATH, Al_2_O_3_ and SiO_2_ with different sizes (from 0.3 μm to 18 μm) and surface modifications (unmodified, modified by the material supplier and *in situ* modified during compounding, using vinyltrimethoxysilane (VTMS) and methyltrimethoxsilane (MTMS)) on the erosion resistance of high temperature vulcanized (HTV) silicone rubber (SR) composites (see Figure 35). The main research focus was on ATH fillers as they have the ability to release water at elevated temperature.

It was found that for very small filler contents, ATH was not favorable for the erosion performance of polymeric composite and by employing 20 wt % of filler, the performance is even worse compared to the base polymer. For a positive effect on this property, a sufficient amount of ATH filler must be added into the polymer matrix. The behavior of the surface modified ground SiO_2_ particles is slightly worse in case of high filler loadings (> 50 wt %), while there was no substantial difference visible at filler loading of 30 wt %. The composite material filled with ground modified ATH of 3.5 µm shows the best performance. The performance of ground modified ATH of 3.5 µm and material supplier filler was very similar [154]. Until the present time, literature on tracking performance of polymer nanocomposites is limited. Experimental investigation onto the tracking resistance of polymer nanocomposites would be of great importance, especially in confirming its performance with nanofillers [159].

#### 6.1.8. Electrical Tree Resistance

The propagation of electrical trees represents one of the major causes for electrical breakdown of high-voltage equipment and power cables insulation [164]. In order to extend the lifetime of an insulation until breakdown, composite materials with barriers and surrounding polymer matrix are commonly used in power engineering. The influence of barriers on the electrical trees propagation were analyzed and many research studies were made on the micro/nanocomposites development for electrical breakdown resistance compared to the base polymer.

Ding *et al.* [165] analyzed the electrical tree growth on samples, such as unfilled Araldite epoxy resin and micro-sized Al(OH)_3_ particle, in different concentrations ranging from 0 to 15 wt %, as reinforcement to enhance the time to breakdown of the composite material. It was concluded that the addition of filler particles in the epoxy resin could make considerable improvements in breakdown resistance by increasing the time of the electrical breakdown with the increase of filler concentrations [165]. Different models could explain the results. For unfilled polymers, it has been assumed that the propagation of electrical trees arises from the formation of a damage process zone (DPZ) which precedes and surrounds the tree tip during the tree growth process [165]. It was supposed that the submicroscopic trees are initiated and grow due to the submicroscopic voids created by thermal fluctuation within the DPZs from the vicinities of the tree tips [165]. With respect to composite materials, it is suggested that by introducing microfillers into the polymer matrix, more submicron-size voids are generated around ATH fillers due to the induced changes in local thermal and residual mechanical stress. Therefore, when a tree channel tip reaches this area, the immovable submicron-voids in the matrix will behave as submicron-trees near the primary tree tip. The DPZ size is then expanded and consequently may increase the resistance to electrical growth of dielectrics and hence increasing the electrical insulation lifetime.

**Figure 36:**
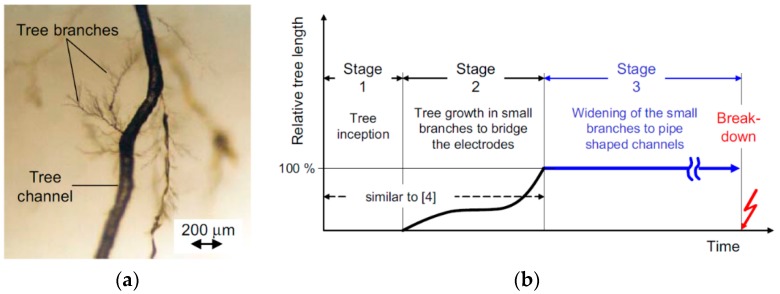
(**a**) Optical micrographs of tree branches and channel and (**b**) stages of electrical tree propagation until the final breakdown (© 2016 IEEE. Reprinted, with permission, from [164]).

Uehara *et al.* [166] analyzed the tree growth and breakdown characteristics of composites based on polymer barrier film layers molded in an EVA copolymer, using a needle – plan configuration and an AC voltage. The electric field was perpendicular to the EVA/barrier film interfaces. It was found that the barrier film retards the electrical tree development, which punctures the film, or develops along the edge of the film. It was also demonstrated that the pressure of the decomposing gas in the tree channels and the interface of the analyzed composite materials play an important role in tree propagation [166]. Vogelsang *et al.* [164] analyzed the tree growth diagrams in composites structure of mica-epoxy winding insulations and their growth characteristic was optically analyzed (see Figure 36a). During the electrical tree propagation, electrical discharges take place in the small branches and their structure changes to hollow pipe-shaped channels (see Figure 36a) [164].

Experimental results showed that the electrical tree propagation could be slowed down when a barrier is introduced between the needle and the plane electrode. This barrier may cause significantly increased time of breakdown values. The enhancement depends on the barrier nature, their thickness and widths, and the dielectric strength of the surrounding polymer interface. When multiple barriers exists, their arrangement influence significantly the time to breakdown and when they are overlapped a much higher time until breakdown is obtained than impinged ones [164]. Based on these results, Vogelsang *et al.* [164] proposed a model that emphasizes the widening of the small branches to pipe-shaped channels. There are three stage in electrical tree propagation until the final breakdown of the composite material (Figure 36b). The first stage represents the tree inception and the second one is the growth of the first small branches to the opposite electrode. The third stage is the stage where the small branches are widened up to pipe shaped channels. It starts when the first branch has reached the opposite electrode and it ends with final electrical breakdown [164]. It can be concluded that the barrier materials and their processing play a major role concerning the time to breakdown of composite insulation materials [164].

Based on similar experimental results, Christantoni *et al.* [167] simulated with the aid of Cellular Automata (CA), the propagation of electrical trees in an insulating system consisted of epoxy resin and mica sheets, which is affected by the applied voltage, the local dielectric strength and the relative permittivity of the involved material. The simulation results indicated that the mica barriers hinder the propagation of electrical trees [167]. Iizuka *et al.* [144] analyzed two types of epoxy/SiO_2_ nanocomposites, Aerosil and Nanopox samples, respectively, in order to clarify the effect of nanofiller dispersion and of coupling agents on electrical tree resistances and voltage endurance. The study revealed that nanofillers and coupling agents could improve treeing resistance in both cases, but depend on the level of nanofiller dispersion [144].

#### 6.1.9. Water Absorption

The presence of absorbed water can have undesired effects on the improvements of electrical properties of micro/nanocomposites and these influences were analyzed by many researchers [87]. Zhang *et al.* [168] analyzed the dielectric behavior in the presence of humidity and dried nanocomposites consisting of either epoxy resin or PE with different concentration of nanoAl_2_O_3_. With respect to the epoxy/Al_2_O_3_ nanocomposites, no difference in the dielectric properties compared to the unfilled polymer were detected under dry conditions. Nevertheless, their dielectric characteristics differed significantly when the adsorbed water amounted to 0.4% b.w. (body weight), which is the normal concentration that occurs under ambient exposure. It can be concluded that the sites for absorbed water in the epoxy nanocomposites increases compared to the unfilled polymer, which does not present interface regions [168]. Zou *et al.* [87] reported that epoxy/SiO_2_ nanocomposites absorbed significantly more water than unfilled polymer due to the fact that extra water was located around the surface of nanoSiO_2_. Epoxy microcomposites were found to absorb less water than the base resin due to the reduced proportion of the polymer in this composite. These hypotheses were confirmed by the measurements on the water uptake, swelling and density change, as a function of humidity. The water shell model, in which percolation of charge carriers occurred through overlapping water shells, explained the low frequency dielectric results [87]. It was observed that nanofillers with functionalized hydrophobic surfaces considerably reduced the amount of water absorbed in the same conditions of humidity.

Dodd *et al.* [169] analyzed the dielectric properties in the presence of humidity for two types of bisphenol-A epoxy resin systems, Araldite CY1301 and Araldite CY1311. It was found that frequency and magnitude parameters, for all the analyzed electrical conduction and dielectric process were dependent on the humidity. In particular, above the glass transition temperature of both epoxy systems, it was found that absorbed moisture was involved in the formation of a bulk quasi-DC dielectric response (QDC) consistent with cluster formation of the absorbed water molecules [169]. Lutz *et al.* [101] analyzed the water absorption behavior of disk-like mineral filled epoxy resin samples determined by charge of weight measurements at different relative air humidity. A significant decrease of volume resistivity was achieved by increasing the water content. This dynamic degradation of the volume resistivity during humidity storage was simulated with a model based on Fick’s diffusion law and the results were verified by periodic measurements of the volume resistivity on epoxy samples during storage at 99% relative humidity [101].

Calebrese *et al.* [158] accomplished a review study of the experimental literature regarding nanodielectrics, such as PAI/nanoAl_2_O_3_ and XLPE/SiO_2_, which indicated numerous inconsistencies in the obtained results. It was concluded that if the processing parameters of the nanocomposites are not carefully selected, the addition of nanoparticles can alter the structure of the materials and can introduce water into the system, generating cavities and facilitating materials degradation [158].

Hui *et al.* [140] analyzed the dielectric behavior of XLPE/SiO_2_ nanocomposites in the presence of humidity. Decreases in AC breakdown strength, increases on losses and space charge formation and significant reduction in water tree aging were obtained. It was also demonstrated that XLPE/SiO_2_ nanocomposites have an increased moisture uptake compared to the base polymer, due to the addition of nano SiO_2_ particles. The results were hypothetically explained by two major factors, which influence the dielectric behavior of XLPE/SiO_2_ nanocomposites, as the concentric water shell around the particles and the charge from inter-particle distances. It was also concluded that a water layer thickness of nanometers tens could initiate percolation in the analyzed XLPE/SiO_2_ nanocomposites [140].

In 2015, Lau *et al.* [53] prepared a review article with the most recent findings and issues concerning nanodielectrics research, highlighting the role of nanofiller/polymer interfaces. One important section referred to the water absorption in nanocomposite materials. It can be concluded that due to the hygroscopic character of nanoparticles, the presence of water on their surface lead to agglomerations. Taking into consideration this aspect, nanoparticles and matrix drying under vacuum conditions or nanoparticles surface functionalization, which replace surface hydroxyl groups and physically block water from getting to the surface of the particles, should be necessary steps. The presence of water can have additional effects on the behavior of nanocomposites, leading to changes in their dielectric behavior [158,170].

### 6.2. Thermal and Mechanical Properties

#### 6.2.1. Thermal Properties

To improve the performance of high-voltage electrical equipment, new electrical insulating materials (with superior properties) are necessary [134]. One important issue is the necessity to use dielectric polymers, as micro/nanocomposites with higher thermal conductivities, which has also excellent processability and low costs. Most of the used polymers are thermally insulating and have a thermal conductivity between 0.1 and 0.5 W/m·K [59]. One of the possibilities to reach this goal is to introduce high-conductivity fillers in polymers, such as Al_2_O_3_, AlN, BN, Si_3_N_4_, beryllium oxide (BeO) or diamond, such as it can be seen in Table 3 [59].

From the all high-thermal conductivity fillers listed in Table 3, only some of them are useful and attractive to be employed in high-voltage applications. Al_2_O_3_ has a relatively high value of λ and is often used as filler because of the low cost and high electrical resistivity. Nevertheless, the main disadvantage in high-voltage applications is its high relative permittivity (ε_r_ ~ 9) [59]. Crystalline SiO_2_ and fumed SiO_2_ are the most commonly material used in electronics and also as fillers to produce highly conductive composites, but their intrinsic thermal conductivity is a bottleneck for the thermal conductivity of their composites [59]. ZnO is a semiconductor and it has been widely exploited as an additive in the rubber industry. Its high thermal conductivity and nonlinear property make its composites useful for electric stress control in high-voltage applications. Another filler with non-linear electrical property and high thermal conductivity is SiC. Due to properties, such as high-saturated carrier drift velocity and high permittivity, its applications in dielectric materials, microelectronic packaging, and high-voltage insulation are limited [59]. Although BeO possesses a higher thermal conductivity than other non-metals and is corrosion resistant, with excellent electrical insulating properties, its toxicity and high cost makes it unattractive for commercial use. Due to the high intrinsic thermal conductivity, low thermal expansion coefficient and high electrical resistivity, AlN attracts much interest. Nevertheless, the low oxidation resistance and relatively high permittivity, limits its applications. Due to its high thermal conductivity and electrical resistivity, low permittivity and density and excellent high temperature resistance, BN is ideal for electronic packaging application. Despite its excellent properties, Si_3_N_4_ has been rarely used as filler for high-thermal-conductivity composites because of its moderate thermal conductivity. Diamond is used as filler for preparing highly conductive composites, but its high cost makes it unattractive for industrial applications. BaTiO_3_ has relatively low thermal conductivity, very high permittivity and density, which makes it unattractive for preparing high-thermal-conductivity composites [59].

**Figure 37:**
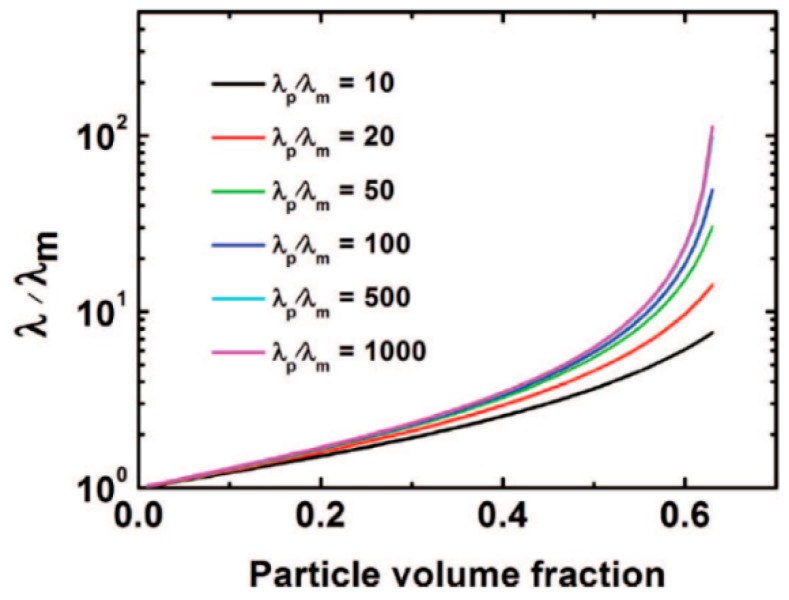
Theoretical prediction of the relative thermal conductivity (λ) of composites (© 2016 IEEE. Reprinted, with permission, from [59]).

The effective thermal conductivity of a composite material composed of one type of filler introduced into a polymer matrix depends on the thermal conductivity of the components, the fillers shape, size and concentration, their dispersion into the polymer and the thermal interfacial resistance [59]. Taking into consideration all these aspects, many theoretical models for computing the thermal conductivity of a composite had been proposed until now, but due to the complex influences (inexact data intrinsic thermal conductivity, shape, size, distribution and orientation of the fillers), only few of them fit the experimental data very well [59]. It was observed that as the intrinsic thermal conductivity of the fillers increases, appears a limit to the thermal conductivity of the composite, as in the theoretical prediction of Nielsen [171] for spherical particles (see Figure 37) with packing fraction 0.637 and the ratios of λ_p_/λ_m_ = 10, 20, 50, 100, 500 and 1000 (λ_p_ is the intrinsic thermal conductivity of the filler and λ_m_ is the thermal conductivity of the composite) [59].

**Figure 38:**
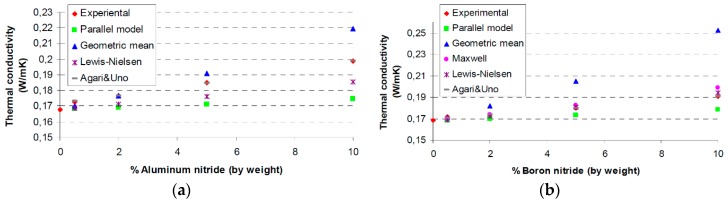
Experimental and predicted thermal conductivity data for (**a**) epoxy resin/AlN and (**b**) epoxy resin/BN composites at 18 °C (© 2016 IEEE. Reprinted, with permission, from [172]).

Kochetov *et al.* [172] compared different theoretical models for predicting thermal conductivity of a two-phase system with experimental data of nanoAlN and nanoBN particles distributed in an epoxy resin matrix. From Figure 38 was concluded that the Agari&Uno model correlated best with the experimental data on the analyzed nanocomposites.

**Figure 39:**
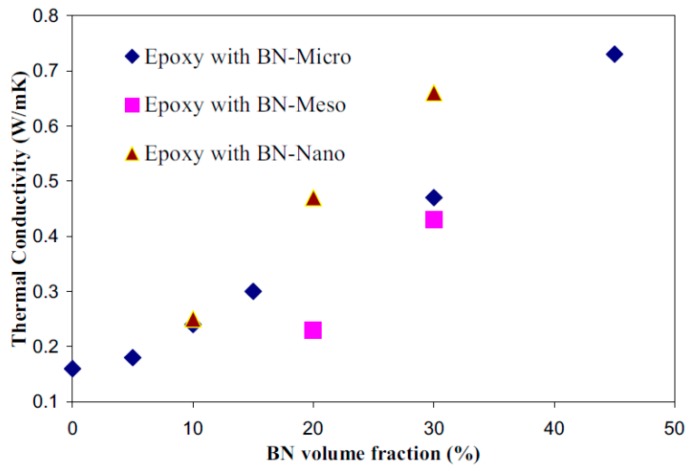
The thermal conductivities of the epoxy composites filled with BN-Micro, BN-Meso and BN-Nano (© 2016 IEEE. Reprinted, with permission, from [173]).

Heat conduction processes in polymer composites are based on phonons. Interfacial thermal resistance is due to the differences between phonon spectra of different phases of the composites and due to scatterings at the interface between these phases [59]. This means that a large interfacial area can cause large phonon scattering and low thermal conductance. Thus, it is expected that the thermal conductivity of polymer composites to increase by increasing the particle size for a given filler loading. Han *et al.* [173] showed in Figure 39 that the thermal conductivities of all analyzed composites increase by increasing BN concentration and that there is no distinctive difference between BN-Micro, BN-Meso and BN-Nano. These results suggest that the size of BN is not necessarily crucial for the thermal conductivity of the epoxy/hardener/filler composites at low to moderate concentrations as the sizes of these BNs are very different [173].

**Figure 40:**
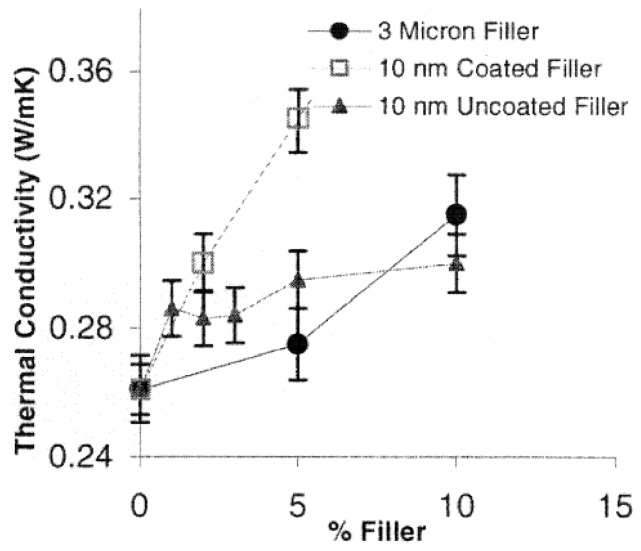
Thermal conductivity in function of the filler concentration characteristics for pure PI, PI microcomposites and PI nanocomposites (nanoparticles uncoated and coated) (© 2016 IEEE. Reprinted, with permission, from [174]).

In general, the high thermal conductivity of a composite is achieved when thermally conductive pathways (percolating network) are formed in the material. A percolating network is obtained if either the filler particles are spherical and their concentration is high, or if the particles exceed a critical value. These requirements increase the material and processing costs and could deteriorate the mechanical and other properties of composite materials [59]. In this situation, there are two methods to ensure a low percolating threshold: (i) immiscible polymer blends can be used as matrix and (ii) composites can be prepared by molding the filler-coated polymer particles [59]. It was reported that the thermal conductivity was enhanced for PI nanocomposite filled with coated nanoparticles compared to pure PI and PI microcomposite (see Figure 40) [174].

**Figure 41:**
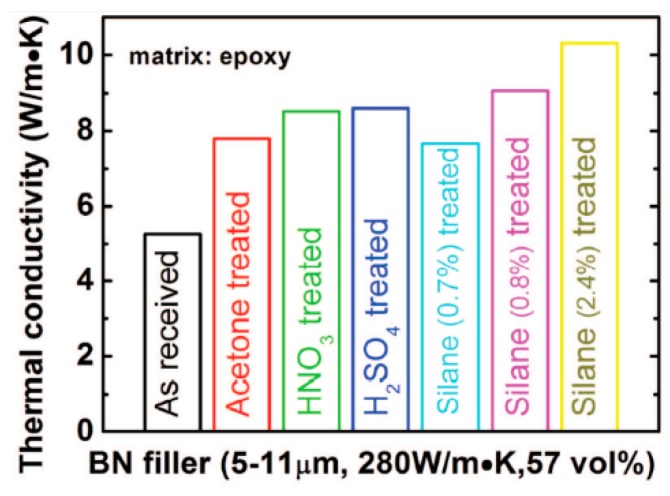
Effect of various surface treatments on the thermal conductivity of epoxy/BN composites (© 2016 IEEE. Reprinted, with permission, from [59]).

The thermal conductivity of nanocomposites cannot be completely determined by size, concentration, dispersion, aspect ratio or particles orientation in the polymer matrix [59]. Xu *et al.* [175] showed in Figure 41 that the surface treatment of BN with acetone, silane, nitric acid (HNO_3_) or sulfuric acid (H_2_SO_4_), resulted in epoxy composites with increased thermal conductivity. The greatest increase was given by silane modification and the least effective was obtained with acetone treated particles.

Thermal conduction in glassy and crystalline dielectrics is caused through elastic vibrations of the crystalline lattice. The use of high thermal conductivity fillers can enhance this process because of the rapidly heat transfer along the fillers [59].

Significant progress has been made during the last few years in the research for high thermal conductivity dielectric micro/nanocomposites. Based on the papers and discussions with numerous equipment manufacturers, there is a strong- interest in improved thermal conductivity with various goals such as: (i) lower operating temperature; (ii) longer service life; (iii) increased operating stress without increasing hot spot temperature; (iv) reduced wall build, *etc.* Various methods have been proposed for improvements ranging from solventless resins to modification of the insulation with high thermal transfer modifiers [176].

#### 6.2.2. Mechanical Properties

Polymer composites used in the production of high-voltage insulating systems, applied in electrical machines, generators, electrical equipment, *etc.*, are often subjected to constant vibrations/abrasion by power frequency magnetic forces and high shear stress under rapid thermal loading conditions [177]. Nevertheless, composite insulation state of the art reports that these mechanical stresses lead to voids/crack initiation or delamination effects with subsequent electrical discharge and catastrophic failure. Many experimental studies were performed on the mechanical properties of composites filled with nano- and micro-fillers and compared with the base polymer.

Yasmin *et al.* [178] investigated composites based on epoxy resin (anhydride-cured diglycidyl ether of bisphenol A, DGEBA) reinforced with 2.5 to 5 wt % graphite. It was concluded that tensile strength and elastic modulus of the composites are increased by adding fillers, and an agglomeration of fillers occurred at 5 wt % fillers concentration. Yang *et al.* [179] studied the mechanical properties of composites based on PP filled with 10, 20, 30 and 40 wt % rice husk flour. They demonstrated that the tensile and impact strength of the composites decreased, while the tensile modulus increased by increasing the filler concentration. In 2005, Lam *et al.* [180] experimentally investigated the mechanical and thermal properties of nano-clay filled epoxy resin composites. The results evidenced that the hardness values of the nanocomposite were increasing by adding nano-clay fillers up to a limit and then were decreasing due to the clusters formed for a high filler concentration. The fracture surfaces observed by microscopic techniques revealed that the size of clusters varied with the nano-clay concentration in the nanocomposite materials. Ray *et al.* [181] analyzed the mechanical properties of vinylester resin matrix composites prepared with 30, 40, 50 and 60 wt % of fly ash. It was found that fly ash enhanced the stiffness and rigidity of composite, but the mechanical strength was reduced at high filler content.

Later, in 2009, Gao *et al.* [182] characterized the mechanical performance of nanocomposites based on PS with nanoCaCO_3_. Tensile and compact tensile tests showed that the strength and toughness of PS were decreasing after the addition of nanoCaCO_3_ particles, which can be explained by the defects induced by interfacial debonding and nanofiller agglomerations. Asi [183] investigated the mechanical properties of Al_2_O_3_-filled glass fiber-reinforced epoxy composites. The results demonstrated that the tensile strength and the shear strength of the composites decreased by increasing Al_2_O_3_ particles content, while the bending strength increased up to 10 wt % filler ratio and decreased at higher ratios.

Panaitescu *et al.* [139] analyzed the influence of nanostructration and fillers dispersion on the mechanical and electrical properties of nanocomposites filled with inorganic fillers, such as SiO_2_ and Al_2_O_3_. An increase of the tensile strength and elongation at break were observed for low nano-oxide concentration (2 wt %). These results were associated with a more favorable dispersion of nanoparticles as well as an increased adhesion at the interface. In 2012, Zaman *et al.* [184] studied the micro- and nano- ZnO-filled i-PP composites with filler concentrations between 2 and 8 wt %. Tensile tests showed that the tensile strength at yield and tensile modulus of the composites tended to increase by increasing contents of microZnO/nanoZnO particles. Nano-filled composites provided improved mechanical properties compared to micro-filled composites for the same filler concentration. It was concluded that the dispersion of particles was optimal at a filler content of 5 wt % since the morphology images and dispersion of nanofillers were better, which led to stronger interfacial adhesion between matrix and fillers. Ibrahim *et al.* [185] investigated composites based on oil palm ash (OPA) as filler in unsaturated polyester, in different concentrations between 10 and 30 vol %. The study revealed that the modulus increases by increasing the filler content, while tensile and flexural strength of UP/OPA composites decrease.

In 2013, Agubra *et al.* [186] analyzed the effects of nano-clay dispersion in glass fiber epoxy composites on the mechanical properties. It was concluded that the high viscosity of the composite generates homogeneity problems due to the agglomeration of the fillers. Chuhan *et al.* [187] investigated the filler size and loading effects on the mechanical and tribological performance of cenosphere-filled vinylester composites. The work revealed that the mechanical and tribological performance could be enhanced, and optimum values were obtained with 6 wt % filler content. Sayer [188] concluded that the elastic modulus and bulking load caring capability of composites based on glass reinforced epoxy resin composites were increased by adding ceramic fillers, such as SiC, Al_2_O_3_, and boron carbide (B_4_C). Sudheer *et al.* [189] studied the mechanical and tribological characteristics of potassium titanate whisker (PTW) reinforced epoxy composites. It was found that PTW additions present beneficiary effect on density, hardness, and stiffness properties of composites; however, mechanical strength and ductility were found to decrease with the increasing content of PTW. In 2015, Pakash *et al.* [190] studied flexural strength, compressive strength, vickers micro-hardness and density for different concentrations of ceria filler. As the content of ceria is increased, an enhancement of the mechanical properties was achieved until a maximum value was reached and then, these properties decreased again by increasing filler concentration.

**Figure 42:**
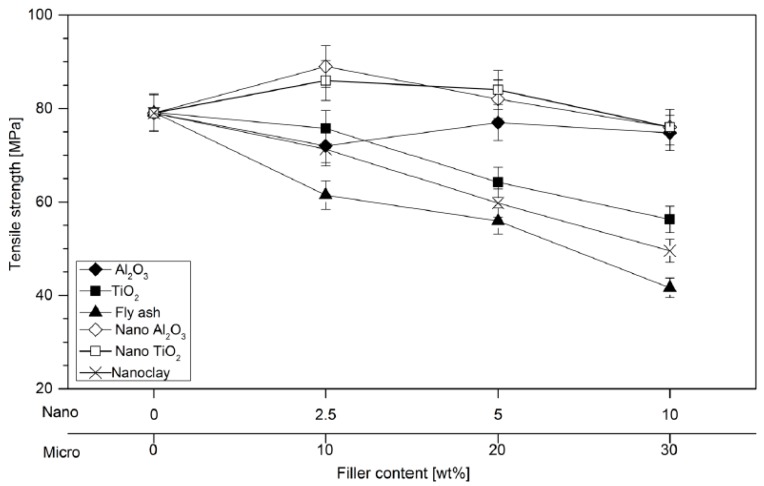
Tensile strength of epoxy resin based micro/nanocomposites *versus* filler content (Reprint with the permission of Strojniski vestnik–Journal of Mechanical Engineering [191]).

Ozsoy *et al.* [191] studied the influence of microfiller (between 10 and 30 wt % Al_2_O_3_, TiO_2_ and fly ash) and nanofillers (between 2.5 and 10 wt % Al_2_O_3_, TiO_2_ and clay) on the mechanical behavior of epoxy based composites. Figure 42 shows the tensile strength *versus* filler content in epoxy resin based micro/nanocomposites. It was observed that the tensile strength of micro-filled composites decreased by increasing filler concentration. In the case of nanocomposite materials, an increase of strength values was observed for up to 2.5 wt % filler content, but beyond this concentration the strength of the composite depleted again.

**Figure 43:**
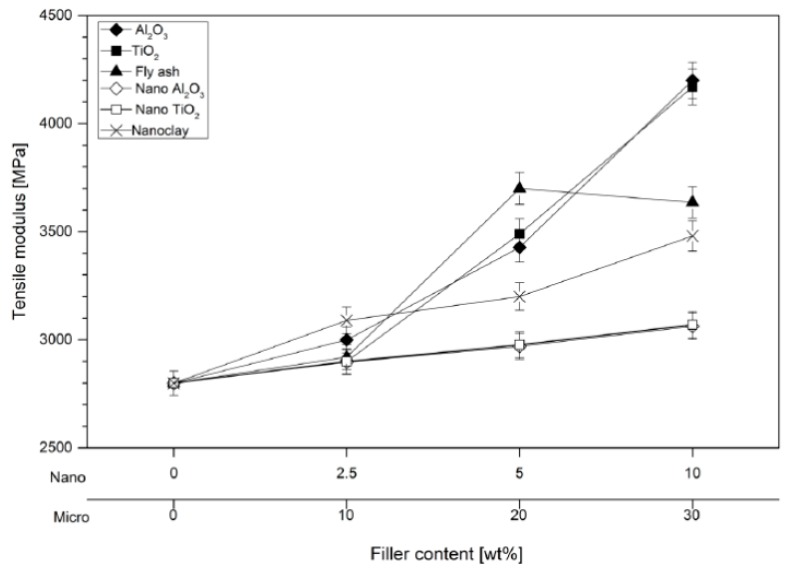
Tensile modulus of epoxy resin based micro/nanocomposites *versus* filler (Reprint with the permission of Strojniski vestnik–Journal of Mechanical Engineering [191]).

**Figure 44:**
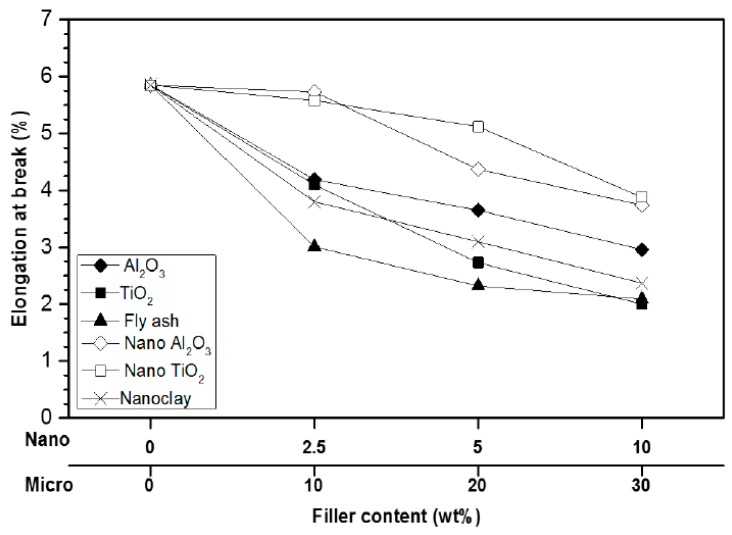
Elongation at break in epoxy resin based micro/nanocomposites *versus* filler content (Reprint with the permission of Strojniski vestnik—Journal of Mechanical Engineering [191]).

Figure 43 illustrates the tensile modulus in dependence on the filler content in epoxy resin based micro/nanocomposites. It was observed that the tensile modulus of epoxy based composites increased with rising filler concentration, which was attributed to the fact that micro- and nanofillers increased the polymer stiffness [191]. Figure 44 describes the elongation at break *versus* the filler content in epoxy resin based micro/nanocomposites. It is clear that the elongation at break decreased by increasing filler concentration distributed into the base epoxy, due to the fact that fillers imposes to the matrix the brittle behavior [191].

Ozsoy *et al.* [191] attributed the decrease in the tensile strength of microcomposites at high filler concentration to the weak adhesion between epoxy matrix and particles. In terms of nanocomposites, the drop in mechanical strength was associated with the inhomogeneous distribution of fillers at higher concentrations, which led to agglomerations and caused stress concentration regions. When it comes to nano-clay-filled epoxy composite even at low filler contents the decrease in strength was related to agglomeration problems [191]. The drop in elongation at break due to the addition of inorganic filler was mainly attributed to the elastic properties of the composite, which depend on the polymer matrix properties. Nevertheless, in the presence of fillers that restrict the mobility of the polymer, epoxy resin shows brittle behavior and the higher the filler content is, the higher the brittleness of the composite is [191].

The attempt of improving the electrical, thermal and mechanical properties of an existing micro- or nanocomposite material or synthesizing a new material can be based on an Edisonian approach. In the case of a new material, many testing samples are necessary to build reliable statistics [94]. The results presented in this review reveal that various nanodielectric systems have a promising future in high-voltage applications.

## 7. Future Trends

Future evolution of polymer based materials in electrical applications will mirror most recent advancements (i) in the preparation and application of new nano-scaled filler (*i.e.*, graphene); (ii) in the design of functional and stimuli-responsive polymer matrix materials (*i.e.*, self-healing concepts) and (iii) in new production techniques (*i.e.*, additive manufacturing). Whilst previous work on polymer based composites is strongly driven by the requirement for materials with enhanced electrical and thermo-mechanical properties that are cheap to produce, advanced material concepts and innovative processing techniques open up the window for completely new product and process designs (*i.e.*, flexible electronics, supercapacitors).

### 7.1. Graphene-Based Nanohybrid Materials

Since the pioneering work of Geim *et al.*, who have successfully identified single layers of graphene in 2004, the one-atom-thick planar sheet of *sp^2^*-bonded carbon atoms have gained enormous attention [192]. Due to its high electrical conductivity, mechanical flexibility, optical transparency, thermal conductivity and low coefficient of thermal expansion graphene has been recently employed in the preparation of polymer based nanocomposites. Several studies reveal that only a very low graphene content is required to enhance the material performance (*i.e.*, high strength and modulus) of polymer based nanocomposites [193,194,195]. The high intrinsic conductivity and 2D structure of graphene favors the formation of a percolation path at low filler loadings since the electrical conduction in a nanocomposite relies on the formation of a continuous conductive network formed by the fillers. Thus, aligned nano-scaled fillers with a high aspect ratio have a high probability to percolate at low filler concentrations in comparison to spherically shaped nanofillers [196].

Current trends in research on nanohybrid materials with graphene give an indication that graphene-based composites play a crucial role in the fabrication of flexible electronics, supercapacitors and energy storage devices [197,198,199]. Moreover, compared to pure graphene the electrochemical stability of graphene-based polymer composites is higher [200]. Sangermano *et al.* [199] have successfully demonstrated the preparation of UV curable epoxy based composites containing evenly distributed graphene platelets. The cured composites were characterized by high *T*_g_ values and improved storage modulus at high temperature [201]. Along with the manufacture of graphene based polymer coatings that comprised a high optical transparency and excellent flexibility, Sangermano *et al.* also developed graphene oxide containing inkjet inks for the fabrication of microelectronic devices [202,203].

Graphene oxide is often used as precursor for the synthesis of processable graphene and is obtained from natural graphite by different methods. The most prominent technique involves the modified Hummers method [204,205]. Graphene oxide surfaces are highly oxygenated and bear various oxygen functionalities (*i.e.*, hydroxyl, epoxide, ketone, and carboxylic groups). In dependence on the surface composition, the solubility of graphene oxide in water and organic solvents can be alterated [193,206,207]. In particular, carbonyl and carboxyl groups located at the edge of the sheets increase the hydrophilicity of graphene oxide and improve its dispersibility in water. For the preparation of graphene, graphene oxide is reduced in a subsequent step by exploiting (photo) chemical and thermal routes [200].

The preparation of epoxy/graphene nanocomposites is a rapidly growing research field, which is reflected by the strong increase in publications in this field [208]. Increased attention is on the preparation of epoxy based graphene composites that comprise improved mechanical performance, enhanced electrical conductivity as well as thermal conductivity. Numerous studies demonstrate that the dispersion of graphene plays an important part in the final properties of the nanocomposites [209].

To tailor the dispersion properties of graphene and graphene oxide in the polymer matrix, several surface modification routes have been established involving nucleophilic substitution reactions (*i.e.*, modification with alkyl amine and dopamine), electrophilic substitution reactions (*i.e.*, functionalization with sulfanilic acid and 4-bromo aniline), condensation reactions (*i.e.*, modification with isocyanates and polyvinyl alcohol) and addition reactions (*i.e.*, functionalization with polyacetylene and aryne) [200]. Along with the chemical surface composition, the preparation procedure governs the dispersion of the graphene platelets in the matrix polymer (solvent mixing *vs*. epoxy impregnation).

Going from the employment of graphene as single filler, current research is geared towards the exploitation of synergistic effects of additional nanofillers in graphene based polymer composites. Particular interest is aimed at binary systems of graphene and carbon nanotubes, which lead to a significant increase in fracture toughness and thermal conductivity [210]. Chemical functionalization of graphene with gold nanoparticles leads to distinctive increase of the electrical conductivity whilst ceramic fillers (*i.e.*, zirconia, gibbsite and boehmite) are applied to improve the capacitive behavior of graphene based epoxy composites [211,212].

### 7.2. Self-Healable Polymer Composites

In recent years, the preparation self-healing polymers that recover their physical and mechanical properties after crack formation or other mechanical damages is also gaining increased attention in the design of functional polymer composites [213]. Healable polymers usually heal in response to a stimulus or an external trigger (*i.e.*, heat, light, and change in pH value) and in principle, two different healing mechanisms are exploited. In terms of autonomically healable polymers, the material properties are regained without an external stimulus whilst with respect to mendable or healable polymers an external stimulus is required to heal [214]. When it comes to the design of healable epoxy based materials, a prominent preparation route involves the application of microencapsulated repair agents. The mechanically rupture of the microcapsules triggers the healing process autonomically and healing of the crack is achieved by the subsequent release of the repair agent [215]. Different repair agents including monomers, hardeners (*i.e.*, polyfunctional thiols), catalysts (*i.e.*, a complex of copper (II) bromide (CuBr_2_) with four 2-methyl imidazol units) are encapsulated to ensure a spatial separation of these reactive components from the bulk material [216,217,218]. These developments of self-healing polymeric materials present interest for electrical insulation systems, which could confer self-healing functionality in a large variety of electrical applications. Particular areas of interest refer to underground power cables and electrical insulation for high-voltage components since failures of the equipment being very costly and, in general, difficult to detect and prevent [219,220,221].

Although the microcapsule approach is characterized by a high versatility and an efficient healing of microscopic cracks, account has to be taken into the fact that both chemical as well as physical properties of the healed zone are not comparable to the bulk material. Moreover, the self-healing properties of the polymer matrix are often limited due to the heterogeneous distribution of the healing agent [213]. To enable a repeated healing of the same damage zone, alternative strategies have been developed that aim at the introduction of reversible crosslinks into polymer materials. By exploiting non-covalent and supramolecular interactions using hydrogen bonds, metal-ligand complexation or self-assembly forces mechanically broken crosslinks can be reformed autonomically [222,223]. To obtain higher mechanical properties, covalent reversible crosslinks based on thermally reversible Diels-Alder/*retro* Diels-Alder chemistry are often used. Prominent examples are polymer materials that undergo concerted [4πs + 2πs] cycloadditions of furan (diene) and maleimide (dienophile) units. In addition, various cyclic dienes such as anthracene or dicyclopentadiene are applied to generate thermally reversible crosslinks with maleimide or bismaleimide moieties [224,225,226,227]. Healing of a crack is achieved by a reheating of the material at temperatures higher than 100 °C under pressure [228,229]. Along with thermally induced healing reactions, [4πs + 4πs] cycloadditions of anthracene and [2πs + 2πs] photocycloaddition reactions of cinnamoyl or coumarin groups are exploited to generate optically healable polymer matrices [230,231,232,233,234]. The ability to reversible bond formation and bond breakage simply by light exposure has gained increased interest since it allows a spatially resolved healing of cracks under ambient conditions.

### 7.3. New Production Routes

#### 7.3.1. Laser Processing of Polymer Composites

Composite materials based on insulating polymeric matrices (*i.e.*, LDPE, high density polyethylene (HDPE), *etc.*) and conducting fillers (*i.e.*, metals, CNTs and nanofibers (CNFs), graphene) are very attractive to be used in numerous applications (*i.e.*, polymeric and flexible electronic, sensor applications, anti-static and electromagnetic interference shielding, *etc.*) due to their good electrical properties determined by the fillers characteristics [235,236]. For concentrations lower than 0.5–5 wt %, composites are characterized by a very low conductivity while it is increasing several orders of magnitude for composites with filler contents higher than the percolation threshold.

In current research, considerable efforts have been made on the fabrication of conducting tracks based on metals and/or carbon on insulating matrices (*i.e.*, thermal and laser treatment of low amount of CNTs and additives and immiscible polymer blends) and laser technique has proven also its utility for *in situ* localized reduction of graphite oxide [237,238]. Cesano *et al.* [235] described the formation of conductive paths obtained by CO_2_-pulsed laser irradiation (laser writing) of MWCNTs in low concentrations and single polymer phase (LDPE, HDPE) composites. Using an investigation at the micro/nano level, conductive paths were successfully generated by a laser induced percolation process. In the irradiated zones, the polymer melting and the formation of an accumulation layer could be clearly shown. In these regions, nanotubes percolation occurs, followed by an enhancement of electrical conductivity with several orders of magnitude. As the layer accumulation is strongly adhering to the undisturbed polymer zone, the obtained tracks are very stable and a control of electrical conductivity along the tracks of any selected pattern is possible. This result is very important in many applications, where the electric signal through the composite must be avoided [235].

#### 7.3.2. 3D Printing

3D printing (3DP) technology is used for the rapid production of 3D objects directly from digital computer aided design (CAD) files [239]. In a 3D printer (3DP) the ink is deposited in thin layers to build up a solid object. A software takes a series of digital cross-sections through a computer aided design (CAD) which are then sent to the 3D printer to achieve different layers according to the 3D printer. After the first layer is complete, the build surface is moved few dozens of micrometers and the second layer is added [239]. The most popular technologies used in low-cost 3D printers are Fused Deposition Modeling (FDM) and Fused Filament Modeling (FFM). In terms of FFM, a thin filament of molten thermoplastic is extruded through a heated nozzle. The filaments cool down and adhere to the layers to build up a solid 3D object [239].

Traditionally, the technology has been used by large companies to produce “rapid prototypes” before production [239]. Nowadays, the technology has found greater appeal in more in the manufacture of final-product across different fields from medical implants to the artistic and creative industries. 3DPs (i.e., RepRap and Fab@Home) have facilitated the manufacturing of customized and producing objects by individualized and personalized approaches. Furthermore, the technology provides low-cost, low-volume and low-risk routes to market for entrepreneurs with novel products leading to a reduction in time to market for innovations [239]. Recent advances in nanotube chemistry enable the dissolution and dispersion of CNTs in various solvents. These developments and research results suggest new alternatives for fabricating CNTs patterns by simply dispersing/printing the dissolved/dispersed particles on substrates. Kordás *et al.* [240] presented a cost-effective and scalable deposition method for generating conductive MWCNTs patterns on paper and polymer surfaces. MWCNTs grown by chemical vapor deposition method (CCVD) were chemically modified to obtain CNTs dispersible in water and the aqueous dispersion was applied on various substrates using a commercial desktop inkjet printer. The electrical behavior of the printed patterns and the process limitations were analyzed. Using 3DP technology, in the last 10 years, groups of researchers and designers worked to create more complex products. One of the future trends in this field involves the incorporation of functional elements (i.e. electronic sensors) into 3D printed macroscale structures. Of course, for this purpose, functional materials and 3D printing methodologies are necessary [240].

## 8. Conclusions

Research and development of composites and nanocomposites materials used in high-voltage applications are challenging. Although much effort has been put forth in the last two decades to investigate the potential electrical benefit of such newly emerging materials and numerous findings in the field were reported, many uncertainties remain unanswered, and much remains to be explored. The tendency in this evolution is towards a multidisciplinary collaboration of electrical, mechanical, thermal engineering, chemistry, material science, physics and other sciences, in order to clarify the fundamental relationship between structure and properties and to bring much more benefits to society with these materials. This close collaboration will hopefully lead to a better understanding of polymers micro/nanocomposites and of the most important component of these materials, respectively the interface region. When all the mechanisms will be identified and clarified, the desired materials with tailored properties proper for high-voltage application will be obtained.

## Figures and Tables

**Figure 24:**
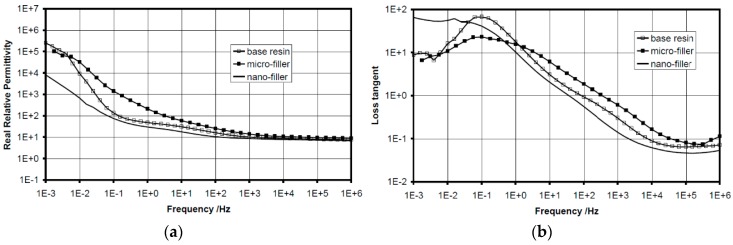
(**a**) Real part of relative permittivity and (**b**) loss tangent of unfilled epoxy resin and epoxy/10 wt % TiO_2_ micro/nanocomposites materials at 393 K (© IOP Publishing. Reproduced with permission. All rights reserved [22]).

**Table 1:** Examples of different types of fillers (Adapted table from [54]).

Origin	Chemical structure	Examples
Natural	Animal	Silk, Wool, Hair
	Mineral	Asbestos
	Cellulose	Wood, Seed, Leaf, Fruit, Stalk, Bast, Grass
Synthetic	Inorganic	Oxides: TiO_2_, SiO_2_, Al_2_O_3_, ZnO, MgO, Sb_2_O_3_
		Hydroxides: Al(OH)_3_, Mg(OH)_2_
		Metals: Al, Au, Ag, B, Sn, Cu, Steel
		Silicates: asbestos, talc, mica, nanoclay, kaolin
		Salts: CaCO_3_, BaSO_4_, CaSO_4_, *etc.*
		Carbides and nitrides: AlN, BN, SiC
	Organic	Carbon and graphite fibers and flakes, carbon nanotubes, carbon black, graphene, graphene oxide
		Natural polymers: cellulose and wood fibers, cotton, flax, starch
		Synthetic polymers: aramid, polyester, polyamide, polyvinyl alcohol fibers

**Table 2:** Examples of different types of nanofillers (Adapted table from [56]).

Nanofiller type	Origins/Structure	Examples
Nano-clay	Phyllosilicates	Kaolinite, Smectite (Talc, Mica, Montmorillonite), Chlorite, Bentonite, Saponite, *etc.*
	PolySilicate	Natural (Magadiite, Ilerite, Zeolite, Silhydrite, Kanemite, Kenyaite) and Synthetic (Zeolite and FluoroHectorite)
	Double Lamellar Hydroxite	Synthetic (Hydrotalcite, *etc.*)
Nano-oxides	Organic	Diatomite;
	Inorganic	NanoTiO_2_, nanoSiO_2_, nanoAl_2_O_3_, nano-antimony-tin oxide (ATO)
Carbon nanotubes (CNTs)	Single-wall	Diameter between 1 and 2 nm;
	Double-wall	Diameter between 2 and 4 nm;
	Multi-wall	Diameter between 4 and 150 nm;
Other nanofillers	Metallic	Nanosilver, nanozinc, nanogold fillers, *etc.*;
	Magnetic	Oxide: ferrites, *etc.*
	Semiconducting	Nano-SiC, nano-ZnO *etc.*

**Table 3:** Thermal conductivities and coefficient of thermal expansion of selected inorganic high-conductivity fillers (Adapted table from [59]).

High-conductivity fillers	Thermal conductivity (W/m·K)	Coefficient of thermal expansion (ppm/°C)
Fused SiO_2_	1.5–1.6	0.4–0.5
Crystalline SiO_2_	3	10
Al_2_O_3_	38–42	7
BeO	300	5.5
ZnO	60	2–3
Si_4_N_3_	86–120	2.7–3.1
BN	29–300	1.1–4.3
AlN	150–220	2.5–5
SiC	85	4.1–4.7
BaTiO_3_	6.2	6
Diamond	2000	0.11–1.23

## Abbreviations

The following abbreviations are frequently used in this manuscript:

AlN: Aluminum Nitride
Al_2_O_3_: Aluminum Oxide or Alumina
Al(OH)_3_: Aluminum Trihydroxide
ATH: Alumina Trihydrate
BN: Boron Nitride
BeO: Beryllium Oxide
BaTiO_3_: Barium Titanate
CaCO_3_: Calcium Carbonate
LS: Layered Silicate
MgO: Magnesium Oxide
SiC: Silicon Carbide
SiO_2_: Silicon Dioxide or Silica
TiO_2_: Titanium Oxide or Titania
ZnO: Zinc Oxide
CNTs: Carbon Nanotubes
SWCNTs: Single Walled Carbon Nanotubes
MWCNTs: Multi-Walled Carbon Nanotubes
GNPs: Graphite Nanoplatelets
PE: Polyethylene
XLPE: Cross-linked polyethylene
LDPE: Low-density polyethylene
HDPE: High-density polyethylene
PP: Polypropylene
i-PP: Isotactic polypropylene
PVC: Polyvinyl chloride
PS: Polystyrene
PUR: Polyurethane
PC: Polycarbonate
PA: Polyamide
PI: Polyimide
PAI: Polyamide-imide (PAI)
EVA: Ethylene-vinyl-acetate
AC: Alternating Current
DC: Direct Current
HVDC: High-Voltage Direct Current
HVAC: High-Voltage Alternating Current
IEC: International Electrotechnical Commission
PD: Partial Discharges
VPI: Vacuum Pressure Impregnation
